# Identification of a novel cuproptosis-related gene signature and integrative analyses in patients with lower-grade gliomas

**DOI:** 10.3389/fimmu.2022.933973

**Published:** 2022-08-15

**Authors:** Jia-hao Bao, Wei-cheng Lu, Hao Duan, Ya-qi Ye, Jiang-bo Li, Wen-ting Liao, Yong-chun Li, Yang-peng Sun

**Affiliations:** ^1^ Hospital of Stomatology, Guanghua School of Stomatology, Sun Yat-sen University, Guangdong Provincial Key Laboratory of Stomatology, Guangzhou, China; ^2^ State Key Laboratory of Oncology in Southern China, Department of Anesthesiology, Sun Yat-sen University Cancer Center, Collaborative Innovation for Cancer Medicine, Guangzhou, China; ^3^ Department of Neurosurgery/Neuro-oncology, Sun Yat-sen University Cancer Center, State Key Laboratory of Oncology in South China, Collaborative Innovation Center for Cancer Medicine, Guangzhou, China

**Keywords:** cuproptosis, lower-grade gliomas, molecular subtypes, tumor microenvironment, immune checkpoint inhibitors, chemoradiotherapy

## Abstract

**Background:**

Cuproptosis is a newly discovered unique non-apoptotic programmed cell death distinguished from known death mechanisms like ferroptosis, pyroptosis, and necroptosis. However, the prognostic value of cuproptosis and the correlation between cuproptosis and the tumor microenvironment (TME) in lower-grade gliomas (LGGs) remain unknown.

**Methods:**

In this study, we systematically investigated the genetic and transcriptional variation, prognostic value, and expression patterns of cuproptosis-related genes (CRGs). The CRG score was applied to quantify the cuproptosis subtypes. We then evaluated their values in the TME, prognostic prediction, and therapeutic responses in LGG. Lastly, we collected five paired LGG and matched normal adjacent tissue samples from Sun Yat-sen University Cancer Center (SYSUCC) to verify the expression of signature genes by quantitative real-time PCR (qRT-PCR) and Western blotting (WB).

**Results:**

Two distinct cuproptosis-related clusters were identified using consensus unsupervised clustering analysis. The correlation between multilayer CRG alterations with clinical characteristics, prognosis, and TME cell infiltration were observed. Then, a well-performed cuproptosis-related risk model (CRG score) was developed to predict LGG patients’ prognosis, which was evaluated and validated in two external cohorts. We classified patients into high- and low-risk groups according to the CRG score and found that patients in the low-risk group showed significantly higher survival possibilities than those in the high-risk group (*P*<0.001). A high CRG score implies higher TME scores, more significant TME cell infiltration, and increased mutation burden. Meanwhile, the CRG score was significantly correlated with the cancer stem cell index, chemoradiotherapy sensitivity–related genes and immune checkpoint genes, and chemotherapeutic sensitivity, indicating the association with CRGs and treatment responses. Univariate and multivariate Cox regression analyses revealed that the CRG score was an independent prognostic predictor for LGG patients. Subsequently, a highly accurate predictive model was established for facilitating the clinical application of the CRG score, showing good predictive ability and calibration. Additionally, crucial CRGs were further validated by qRT-PCR and WB.

**Conclusion:**

Collectively, we demonstrated a comprehensive overview of CRG profiles in LGG and established a novel risk model for LGG patients’ therapy status and prognosis. Our findings highlight the potential clinical implications of CRGs, suggesting that cuproptosis may be the potential therapeutic target for patients with LGG.

## Introduction

Lower-grade gliomas (LGGs; addressed as WHO grades II and III here), consisting of diffuse low-grade gliomas and intermediate-grade gliomas, are usually slow-growing, infiltrative, and intermittently progressive, which accounts for approximately 22% of all brain tumors in adults (1). Most LGGs can be further divided according to their clinical histopathologic features and classic molecular markers, including isocitrate dehydrogenase (*IDH*) mutation and the 1p/19q codeletion status (2). With tremendous progress that has been made in therapy like surgical resection and chemotherapy, LGGs often have better prognoses, while high-grade gliomas (HGG) have worse prognoses due to their malignant aggressivity (3). However, although 5-year overall survival (OS) for patients with LGG is 85%, progression-free survival (PFS) for those with unresectable/residual disease requiring treatment is approximately 40%, making the prognosis grim (4). Meanwhile, it was indicated that progression of LGGs occurs in almost 70% of patients within 10 years, thus worsening the prognosis (5). Hence, there is an urgent need to characterize specific and practical molecular signatures for the accurate diagnosis, individualized treatment, and assessment of the prognosis of LGG.

Cuproptosis, first proposed by Todd R. Golub’s lab in 2022, is a unique non-apoptotic programmed cell death distinguished from known death mechanisms like ferroptosis, pyroptosis, and necroptosis (6). It is copper-triggered and mediated by protein lipoylation mainly in mitochondria. Mechanistically, cuproptosis occurs through the direct binding of copper to the lipoylated components of the tricarboxylic acid (TCA) cycle. When respiring, the lipoylated TCA enzymes [particularly the pyruvate dehydrogenase (PDH) complex] increase and result in an abnormal aggregation of lipoacylated proteins and the loss of Fe-S cluster–containing proteins, which leads to inevitably acute proteotoxic stress and ultimately cell death (6). The research provided a further in-depth look at the role of copper and mitochondria homeostasis, demonstrating a potentially critical role of cuproptosis in cell biology (7).

Numerous recent studies have consistently illuminated the functions of copper homeostasis and mitochondria in many diseases, including heart failure, neurodegenerative diseases, metabolic diseases, and genetic disorders (8). Copper serves as a catalytic and structural cofactor for enzymes that regulate mitochondrial respiration, antioxidant defense, redox signaling, kinase signaling, autophagy, and other processes (9–11). It can also function as a signal to enable responses to the enhanced host defenses resulting from immune activation (12). It has been an excellent candidate for cancer treatment since the 1960s. Emerging studies have shown that an elevated level of copper is directly associated with cancer progression. Brady et al. demonstrated that copper is critical in driving lung adenocarcinoma *via* regulating the autophagic kinases *ULK1/2* (13). Mittal and his colleagues found that the depletion of mitochondrial copper significantly suppresses triple-negative breast cancer in mice (14). The inhibition of copper trafficking can attenuate cancer cell proliferation (15). Meanwhile, mitochondria are critical in various cellular functions such as cellular energy metabolism, ion homeostasis regulation, redox signaling, and cell death, and its dysfunction has already been known in glioma initiation, progression, and drug resistance (16–18). Different levels of mitochondrial aberrations might contribute to disparities in the aggressiveness of patients with glioma. Yang et al. demonstrated that mitochondrial *PKM2* plays a vital role in the ROS adaptation of cancer cells, which implicates the *HSP90-PKM2-Bcl2* axis as a potential target for therapeutic intervention in gliomas (19). The suppression of mitochondrial ROS can also drive the glioma therapeutic resistance due to the dysregulation of glioma stem-like cells (20), while the activation of the mitochondrial-dependent apoptotic pathway potentiates temozolomide sensitivity and thus improves patients’ outcomes (21). These discoveries shed light on the tumor copper and mitochondria homeostasis related to cuproptotic plasticity and possibly explain whether and how cuproptosis is associated with the persistence, differentiation, and expansion of cancer cells. It is a conceivable complex interplay that cuproptosis would be a new molecular signature and target for future investigations. The regulations of the cancer cells’ susceptibilities to cuproptosis should be a fruitful area in cancer research. However, to our best knowledge, it remains to be elucidated whether cuproptosis plays a critical role in LGG, and the relationships with survival in LGG patients have never been explored.

In this study, we aim to investigate the whole aspects of cuproptosis-related genes (CRGs) and their values in the prognosis, tumor microenvironment (TME) infiltration, and responses to treatments in LGG through integrative bioinformatics analyses. The results were further verified in clinical specimens from Sun Yat-sen University Cancer Center (SYSUCC) through quantitative real-time PCR (qRT-PCR) and Western blotting (WB).

## Materials and methods

### Data acquisition

The gene expression data and corresponding clinical information of LGG samples were obtained from The Cancer Genome Atlas (TCGA) (https://portal.gdc.cancer.gov/) and Chinese Glioma Genome Atlas (CGGA) (http://cgga.org.cn/) databases. A total of 529 samples with a gene expression profile, copy number variation (CNV), single-nucleotide variant (SNV), and relevant clinicopathological data were downloaded from TCGA-LGG. The fragments per kilobase million values of TCGA-LGG were transformed into transcripts per kilobase million. For all the included RNA-seq data, normalization and log2 transformation were performed. The loss and gain levels of copy-number changes have been identified using segmentation analysis and the GISTIC algorithm. The SNV data were further analyzed by R package “maftools” and visualized by R package “oncoplot”. We further downloaded the TPM- normalized GTEx RNAseq data of 1,152 normal human brain samples from the GTEx data portal (https://xenabrowser.net/datapages/). Two CGGA cohorts that contained 182 and 174 LGG samples (CGGA1, mRNAseq_325, RNA-seq; CGGA2, mRNA-array_301, Microarray) were obtained as external validation cohorts.

### Unsupervised clustering for cuproptosis-related genes

A total of 13 CRGs were extracted from the previous study (6), and the details of genes were shown in **Supplementary Table 1**. Based on the expression levels of CRGs, we performed consensus unsupervised clustering analysis to classify patients into distinct cuproptosis-related clusters (CRG clusters) using the R package “ConsensusClusterPlus” (22), with the parameters of reps = 1000 and pItem = 0.8. Principal component analysis was conducted to show the classification of CRG clusters. Then, we compared the OS probability of CRG clusters using the R package “survival” and “jskm”. A landmark time of 9 years was chosen. Chemoradiotherapy sensitivity–related genes (CRSGs) and immune checkpoint genes (ICGs) were further retrieved (23–28), and their expression levels between CRG clusters were analyzed.

### Estimation of tumor microenvironment cell infiltration between cuproptosis-related gene clusters

R package “ESTIMATE” can calculate TME scores including the stromal score, immune score, and estimate score using gene expression profiles (29). TME scores for LGG patients were evaluated and compared between CRG clusters. A single sample gene-set enrichment analysis (ssGSEA) algorithm was used to quantify the immune infiltration degree of immune cells in the LGG TME.

### Gene set variation analysis and gene set enrichment analysis

To investigate the difference of the biological function between CRG clusters, gene set variation analysis (GSVA) was performed with “c2.cp.kegg.v7.5.symbols” and “c5.go.bp.v7.5.symbols” using R package “GSVA”. R package “pheatmap” was applied to visualize the results. GSEA was performed by R package “clusterProfiler” to determine whether the prior-defined functional sets of genes differ significantly between CRG clusters with the hallmark gene set (“h.all.v7.2.symbols”) from the MSigDB database.

### Identification of differentially expressed genes between cuproptosis-related gene clusters and functional annotation

The differentially expressed genes (DEGs) between different CRG clusters were identified using R package “Limma”. The significance criteria for identifying DEGs was set as |log2 (FoldChange)| > 0.5 and adjusted *P*-value< 0.05. To explore the biological functions of CRG cluster-related DEGs, Gene Ontology (GO) and Kyoto Encyclopedia of Genes and Genomes (KEGG) enrichment analyses were conducted by applying the “clusterProfiler” package (30).

### Identification of cuproptosis gene clusters in lower-grade glioma

We performed univariate Cox regression analysis for CRG cluster–related DEGs to identify DEGs that were related to OS (OS-related DEGs). According to the expression levels of OS-related DEGs, we performed consensus unsupervised clustering analysis using R package “ConsensusClusterPlus”, with the parameters of reps = 1000 and pItem = 0.8. The TCGA-LGG patients were divided into distinct cuproptosis gene clusters, and the OS time was compared through Kaplan–Meier analysis.

### Construction of the cuproptosis-related prognostic model

Then, patients in the TCGA-LGG cohort were randomly divided into the training cohort and internal testing cohort at a ratio of 1:1 using R package “caret”. Based on OS-related DEGs, the least absolute shrinkage and selection operator (LASSO) Cox regression were performed to reduce the dimension of high-latitude data using R package “glmnet”. Ten-fold cross-validation was employed to avoid the overfitting problem and select the penalty parameter (λ) according to the minimum criteria. We conducted a multivariate Cox regression analysis to determine genes from candidate genes and further performed GSEA analyses based on a single gene expression, respectively. Next, we construct the cuproptosis-related predictive model in the training cohort. We calculated the CRG score for each sample using the following formula: $CRG\_score=\sum_{i=1}^{n}Coef_{i}\times Exp_{i}$ , with Coef indicating the coefficient and Exp referring to the expression level of each CRG.

### Evaluation and validation of the cuproptosis-related prognostic model

The prognostic scoring system for LGG patients was established, and the median value of the predicted CRG scores was regarded as the cut-off. Then, patients were divided into high-risk (CRG score > median value) and low-risk (CRG score< median value) groups accordingly. R package “survival” and “survminer” was applied to compare the survival probability between the two groups *via* Kaplan–Meier analysis. The R package “timeROC” was employed to perform 1-, 3- and 5- year receiver operating characteristic (ROC) analysis and calculate the value of the area under the curve (AUC). The calibration plots were further conducted to better validate the advantage of the CRG_score. The expression levels of CRGs were analyzed between different CRG score groups. The internal testing cohort and two external cohorts CGGA1 and CGGA2 were employed to verify the cuproptosis-related prognostic model. The CRG score was calculated for LGG patients in each cohort, and samples were divided into different risk groups. Similarly, they were subjected to Kaplan–Meier analysis, ROC analysis, and calibration analysis. In CGGA1 and CGGA2 cohorts, patients were also stratified into four risk-treatment subgroups by the CRG score and treatment with temozolomide (TMZ) or radiotherapy. In addition, survival analyses among risk-treatment subgroups were conducted.

### Correlations of cuproptosis-related gene score with immune infiltrates and cancer stem cell index in lower-grade glioma

In order to identify the gene sets of statistical differences between high- and low-risk groups, GSEA was performed. The annotated gene sets “h.all.v7.2.symbols” and “c5.bp.v7.2.symbols” from the MSigDB database were adopted in our analysis. The enrichments of gene sets with an adjusted *P*-value<0.05 were regarded to be significant. We employed R package “ESTIMATE” to evaluate the TME score levels between high- and low-risk groups. Cell-type Identification by Estimating Relative Subsets of RNA Transcripts (CIBERSORT) is a developed algorithm that uses a set of reference gene expression matrices to evaluate 22 immune cell type proportions from bulk tumor sample expression data based on the principle of linear support vector regression (31). We processed the TCGA-LGG RNA-Seq data (TPM normalized) to calculate the relevant abundance of immune cells. We analyzed the Spearman correlation between the abundance of infiltrating immune cells and the CRG score. Furthermore, we downloaded the RNAss file named “StemnessScores_RNAexp_20170127.2.tsv”. The tumor stem cell characteristics were extracted from the transcriptome and epigenetics of the samples and then used to evaluate the stem cell-like features of tumors. We performed a correlation analysis the investigate the association between the CRG score and Cancer Stem Cell (CSC) index.

### Correlations of cuproptosis-related gene score with tumor mutation burden and immune checkpoint genes in lower-grade glioma

TMB and ICGs were associated with patients’ response rate to immunotherapy. We extracted the mutation annotation format (MAF) from the TCGA database with the “maftools” R package to identify the mutational landscape of LGG patients between different CRG score groups. The TMB score was also calculated for each LGG patient in the entire TCGA cohort. Then, we evaluated the correlations of ICGs with the CRG score and five genes in the cuproptosis-related prognostic model using Spearman’s rank correlation coefficient.

### Estimation of cuproptosis-related prognostic model in immunotherapy response

The immunotherapy response for LGG patients was estimated through the Tumor Immune Dysfunction and Exclusion (TIDE) algorithm (http://tide.dfci.harvard.edu), which can help doctors select patients who are more suitable for immunotherapy (32). Furthermore, GSE126044, GSE78220, checkmate cohort (33), and IMvigor210 cohort (34) were used to validate the predictive ability of the cuproptosis-related prognostic model in immunotherapy response.

### Correlations of cuproptosis-related gene score with cuproptosis-related genes and chemotherapeutic sensitivity in in lower-grade glioma

We compared the expression levels of CRSGs between different risk groups and performed the correlation between CRG scores and gene expression levels. The calcPhenotype function of the “oncoPredict” R package was applied to estimate drug sensitivity scores for common drugs in the LGG therapy regimen including TMZ, procarbazine, teniposide, and vincristine in the TCGA cohort. The lower-imputed drug sensitivity represents more sensitivity to the drug.

### Independent prognostic analysis and establishment of a nomogram

We obtained the clinical characteristics including the age, grade, and IDH mutation status of LGG patients in the entire TCGA cohort and two CGGA cohorts. In combination with the CRG score, these variables were analyzed in univariate and multivariable Cox regression analyses.

To individualize the predicted LGG patients’ survival probability, we developed a nomogram using clinical characteristics and the CRG score. The R package “rms” and “regplot” were employed. Time-dependent ROC analysis was conducted to assess the predictive accuracy for 1-, 3-, and 5-year survival probability. The calibration plots were applied to compare model-predicted probability with observed outcomes in the TCGA-LGG cohort and two validation cohorts (CGGA1 and CGGA2).

### Tissue samples, quantitative real-time PCR, and Western blotting

To further validate the potential roles of signature genes in LGG, five paired LGGs and matched normal adjacent tissue samples were collected from the SYSUCC. Ethical approval was confirmed by the ethical committee of the hospital. Associated clinicopathological features were further confirmed as listed in **Supplementary Table 2**. Tissue specimens were frozen in liquid nitrogen and stored at –80°C until used.

Total RNA was extracted with a TRIzol Reagent (ThermoFisher: #15596018), and the concentration was calculated by the A260/A280 ratio. The PrimeScript RT reagent kit (EZBioscience: #A0010CGQ) and SYBR-Green PCR reagent (EZBioscience: #A0012-R2-L) were used to perform cDNA synthetization and further conduct RT-qPCR based on the LightCycler ^®^ 480 System (Roche). The housekeeping gene GAPDH was used as an endogenous control. The 2^−ΔΔCT^ cycle threshold method was used to calculate the relative expression. **Supplementary Table 3** lists the primers used in this study.

The protein expression levels of crucial CRGs were confirmed by Western blotting. Tissues were treated with RIPA lysis buffer (Fdbio: #FD009) containing phosphatase and protease inhibitors. The BCA protein detection kit (ThermoFisher: #23227) was applied to detect the protein concentration. Equivalent protein was then separated by 10% Tris-Tricine SDS-PAGE and transferred onto polyvinylidene fluoride (PVDF) membranes. After blocking with skimmed milk in TBST for 2 h, the membrane was further probed using antibodies against GAPDH (Proteintech: #60004-I-Ig), C21orf62 (Signalway Antibody, SAB: #C08364H), DRAXIN (Proteintech: #26342-1-AP), ITPRID2 (Proteintech: #14157-1-AP), MAP3K1 (Proteintech: # 19970-1-AP), and MOXD1 (Bioss: bs-17733R) overnight at 4°C. The membranes were subsequently washed with Tris-buffered saline containing Tween and then incubated with an HRP‐conjugated anti-rabbit antibody at 37°C for 1 h. Finally, the bands on the membranes were observed with a ChemiDoc™ Imaging System.

### Statistical analysis

All statistical analyses were performed using R software (Version 4.1.2). Correlation coefficients were evaluated by Spearman analysis. To compare variables between two groups, we employed the independent sample t-tests for normally distributed continuous variables, and Mann–Whitney U tests for non-normally distributed continuous variables. One-way ANOVA and Kruskal–Wallis tests were used to perform the difference comparisons of three or more groups. The survival analysis was conducted *via* the Kaplan–Meier method, and log-rank tests were employed to identify the significance of differences. The statistical significance was defined as *P*< 0.05.

## Results

### Landscape of genetic and transcriptional variations of cuproptosis-related genes in lower-grade glioma

**Figure 1:**
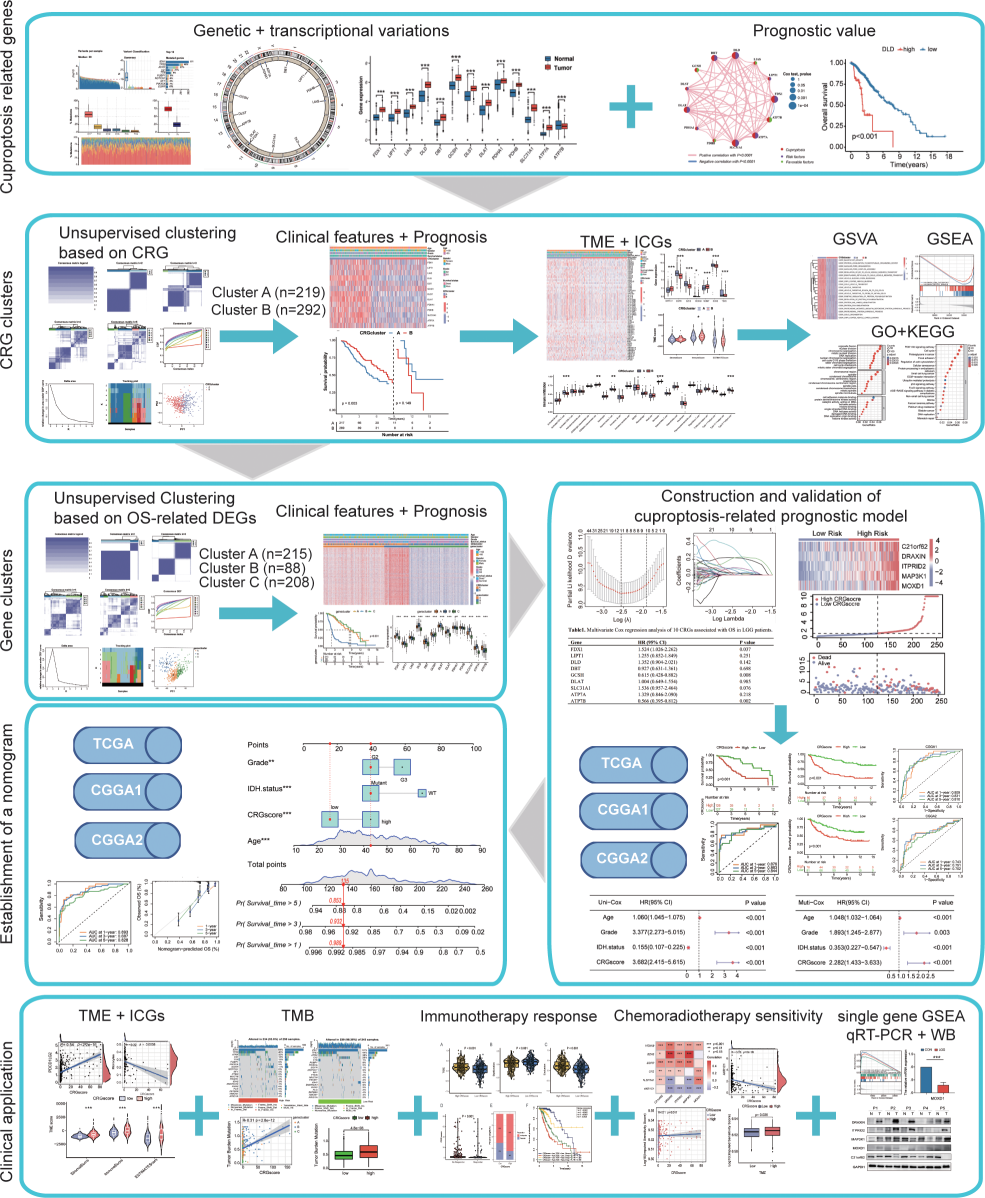
Flow chart of this study. *P< 0.05; **P< 0.01; ***P< 0.001.

**Figure 2:**
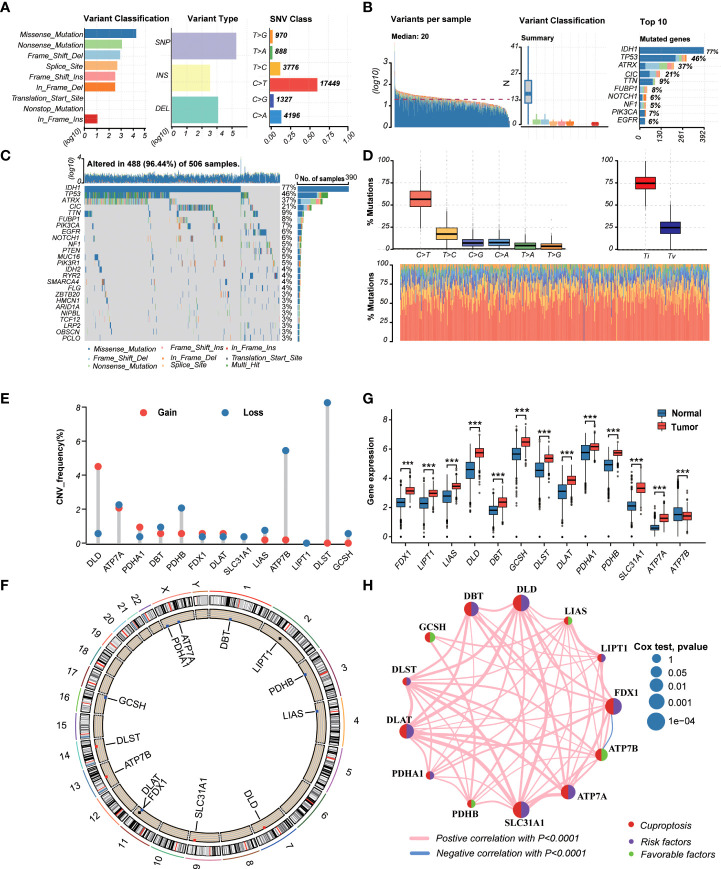
Landscape of genetic and transcriptional variations of cuproptosis-related genes (CRGs) in lower-grade glioma (LGG). **(A, B)** Summary of variation across 506 lower-grade glioma (LGG) patients including the variant classification, variant type, single-nucleotide variant (SNV) class, variants per sample, and top 10 mutated genes. **(C, D)** Landscape of genetic variations of 506 LGG patients in The Cancer Genome Atlas (TCGA) cohort. **(E)** Copy number variation (CNV) amplifications and deletions of CRGs in LGG patients. **(F)** The circus plot showed the location of CNV alteration of CRGs on 23 chromosomes. **(G)** Differences in the expression levels of 13 CRGs between tumor and normal samples. (tumor, red; normal, blue) *P-*values were shown as: ***P< 0.001. **(H)** The network showed interactions among CRGs in LGG. LGG, lower-grade glioma (WHO II and III); TCGA, the Cancer Genome Atlas; SNV, single-nucleotide variant; CNV, copy number variation; CRGs, cuproptosis-related genes.

TThe workflow of the study is outlined in **Figure 1**. A total of 13CRGs were included in our study The genetic mutation landscape in LGG patients is shown in **Figures 2A–D**. Of the 506 LGG patients in the TCGA cohort, 488 (96.44%) had genetic mutations and *IDH1* had the highest mutation frequency (77%), followed by *TP53*, *ATRX*, *CIC*, and *TTN*. However, only seven samples had genetic mutations in CRGs (**Supplementary Figure 1**). We investigated the frequencies of the CNVs of 13 CRGs in LGG. *DLD* exhibited the highest amplification frequency, while *ATP7B* and *DLST* had a widespread frequency of CNV loss (**Figure 2E**). **Figure 2F** shows the location of the CNV alterations of 13 CRGs on 23 chromosomes.

We then explored the expression levels, molecular interactions, and prognostic values of 13 CRGs. Twelve CRGs were upregulated in tumor samples including *FDX1*, *LIPT1*, *LIAS*, *DLD*, *DBT1*, *GCSH*, *DLST*, *DLAT*, *PDHA1*, *PDHB*, *SLC31A1*, and *ATP7A* (*P*< 0.001), whereas only *ATP7B* was downregulated (*P*< 0.001) (**Figure 2G**). **Figure 2H** exhibits the molecular interactions between CRGs. Nine prognostic CRGs were identified by Kaplan–Meier analysis and univariate Cox regression analysis (**Supplementary Figure 2**). The result of multivariate Cox regression analysis further revealed that three prognostic CRGs (*FDX1*, *GCSH*, and *ATP7B*) were independent prognostic factors (**Table 1**).

**Table:** Table 1. Multivariate Cox regression analysis of 10 cuproptosis-related genes associated with overall survival in lower-grade glioma patients.

Gene	HR (95% CI)	P-value
FDX1	1.524 (1.026-2.262)	0.037
LIPT1	1.255 (0.852-1.849)	0.251
DLD	1.352 (0.904-2.021)	0.142
DBT	0.927 (0.631-1.361)	0.698
GCSH	0.615 (0.428-0.882)	0.008
DLAT	1.004 (0.649-1.554)	0.985
SLC31A1	1.536 (0.957-2.464)	0.076
ATP7A	1.329 (0.846-2.090)	0.218
ATP7B	0.566 (0.395-0.812)	0.002

### Identification of cuproptosis-related gene clusters in lower-grade glioma

In order to investigate the expression features and potential biological characteristics of CRGs in LGG, a consensus clustering algorithm was utilized to classify LGG patients in the TCGA cohort. Based on the expression of 13 CRGs, patients were categorized into CRG cluster A (n=219) and CRG cluster B (n=292) (**Supplementary Figures 3A**–**3H**). The PCA plot demonstrated an obvious different distribution between CRG clusters (**Supplementary Figure 3I**).

### Correlations of cuproptosis-related gene clusters with clinical features, chemoradiotherapy sensitivity–related genes, immune checkpoint genes and tumor microenvironment

**Figure 3:**
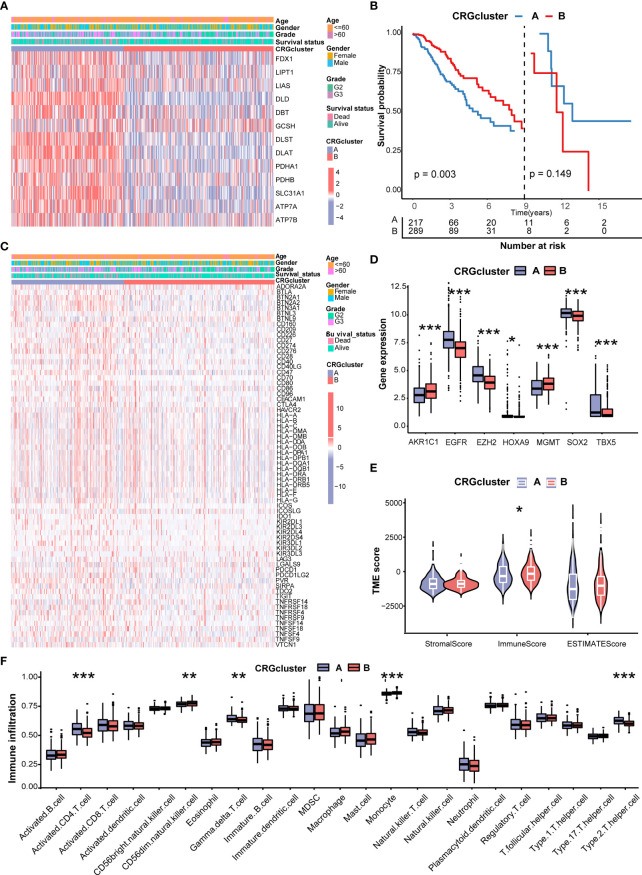
Correlations of CRG clusters with clinical features, CRSGs, immune checkpoint genes (ICGs), and tumor microenvironment (TME). **(A)** The heatmap showed the different expressions of CRGs and clinicopathological characteristics between CRG cluster A and **(B)**(B) Landmark survival analysis for two CRG clusters. The overall survival probability of LGG patients in the two CRG clusters was calculated by Kaplan–Meier analysis (log-rank tests). A landmark time of 9 years was set. **(C)** The heatmap showed the different expressions of ICGs and clinicopathological characteristics between CRG cluster A and B **(D)** Differences in the expression levels of seven chemoradiotherapy sensitivity–related genes (CRSGs) between CRG cluster A and B**(E)** Correlations between the two CRG clusters and TME scores. **(F)** Differences in the abundance of infiltrating immune cells between CRG cluster A and B (CRG cluster A, blue; CRG cluster B, red) *P* values were shown as: *P< 0.05; **P< 0.01; ***P< 0.001. CRGs, cuproptosis-related genes; CRSGs, chemoradiotherapy sensitivity–related genes; ICGs, immune checkpoint genes; TME, tumor microenvironment; LGG, lower-grade glioma (WHO II and III).

**Figure 3A** shows the different expressions of CRGs and clinicopathological characteristics between CRG cluster A and B. CRG cluster A was preferentially associated with higher expression levels of CRGs, higher grade (G3), and more death events. As the Kaplan–Meier survival curves crossed, we employed landmark analysis to compare the difference between CRG clusters (**Figure 3B**). The result of landmark analysis showed a longer OS in LGG patients in CRG cluster B within 9 years (*P* = 0.003). Nevertheless, no significant difference was found in the survival probability beyond 9 years (*P* = 0.149).

Then, we explored the correlation of CRG clusters with ICGs, CRSGs, and the TME. We found that CRG cluster A was associated with a higher expression of ICGs (**Figure 3C**). In addition, CRSGs, including *AKR1C1*, *EGFR*, *EZH2*, *HOXA9*, *HGMT*, *SOX2*, and *TBX5*, were differentially expressed between two CRG clusters (**Figure 3D**). To explore the potential function of CRGs in the immune infiltration of LGG, we compared the TME score and the relevant abundance of immune cells between two CRG clusters using “ESTIMATE” and “ssGSEA” algorithms. Patients in CRG cluster B had higher immune scores than those in CRG cluster A (**Figure 3E**). We observed that the immune infiltration levels of the activated CD4+ T cell, gamma delta T cell, and Type 2 T helper cell were significantly higher in CRG cluster A than those in CRG cluster B, while the CD56 dim natural killer cell and monocyte had significantly lower infiltration levels in CRG cluster A than those in CRG cluster B (**Figure 3F**).

### Identification of differentially expressed genes between cuproptosis-related gene clusters and functional annotation

**Figure 4:**
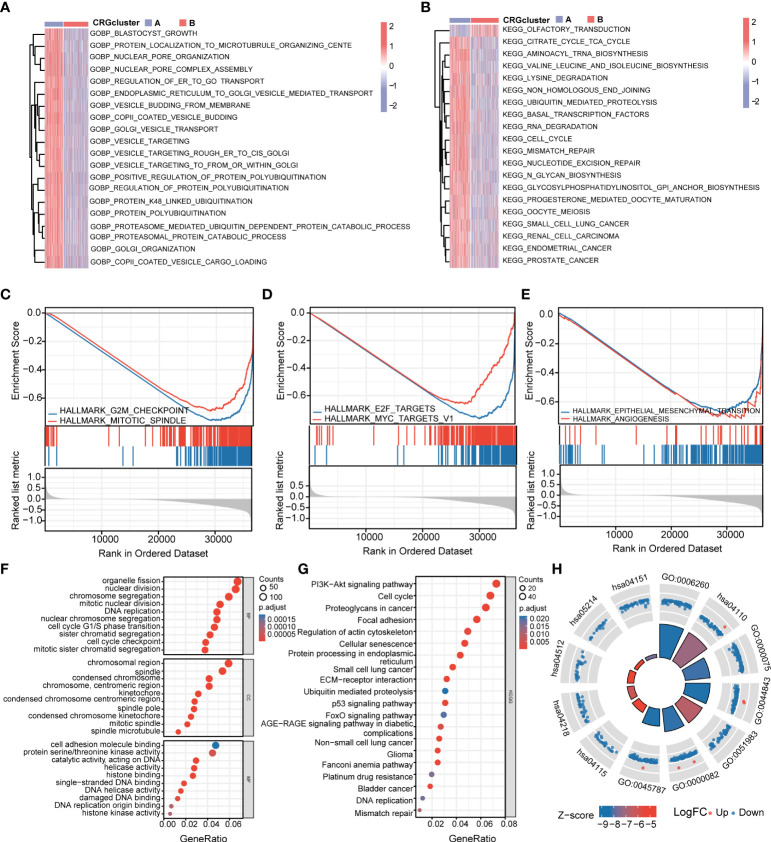
Functional enrichment analysis and identification of differentially expressed genes (DEGs) between CRG clusters. **(A)** Gene set variation analysis (GSVA) of gene ontology biological process (GOBP) terms between CRG cluster A and B, in which red and blue represent activated and inhibited, respectively. **(B)** GSVA of Kyoto Encyclopedia of Genes and Genomes (KEGG) terms between CRG cluster A and B, in which red and blue represent activated and inhibited, respectively. **(C-E)** Gene set enrichment analysis (GSEA) of significant HALLMARK enriched in CRG cluster **(A) (F–H)** GO and KEGG enrichment analyses of DEGs between two CRG clusters. DEGs, differentially expressed genes; GSVA, gene set variation analysis; GOBP, gene ontology biological process; GSEA, gene set enrichment analysis; GO, Gene Ontology; KEGG, Kyoto Encyclopedia of Genes and Genomes.

To further explore the functional annotation between CRG cluster A and B, GSVA and GSEA were performed. The results of the GSVA of the gene ontology biological process (GOBP) showed that CRG cluster A was significantly enriched in transportation-related processes including Golgi vesicle transport, nuclear pore organization, and vesicle targeting (**Figure 4A**). In addition, the GSVA of KEGG terms showed that CRG cluster A was abundant in metabolism-related pathways (citrate cycle TCA cycle, glycosylphosphatidylinositol GPI anchor biosynthesis), cancer-related pathways (small cell lung cancer, endometrial cancer), cell cycle–related pathways (cell cycle), and genomic stability–related pathways (mismatch repair, nucleotide excision repair) (**Figure 4B**). GSEA indicated that CRG cluster A was predominantly associated with the cell cycle, tumorigenesis, and metastasis including the G2M checkpoint hallmark, E2F target hallmark, epithelial mesenchymal transition hallmark, and angiogenesis hallmark (**Figures 4C–E**) (**Supplementary Tables 4**–**5**).

A total of 1,966 CRG cluster–related DEGs were identified between CRG clusters using R package “Limma”. Consistent with GSVA and GSEA, the result of GO and KEGG showed that these DEGs were associated with the cell cycle, genomic stability, and cancer, which revealed that cuproptosis plays a significant role in tumorigenesis and metastasis (**Figures 4F–H**) (**Supplementary Table 6**).

### Identification of cuproptosis gene clusters in lower-grade glioma

**Figure 5:**
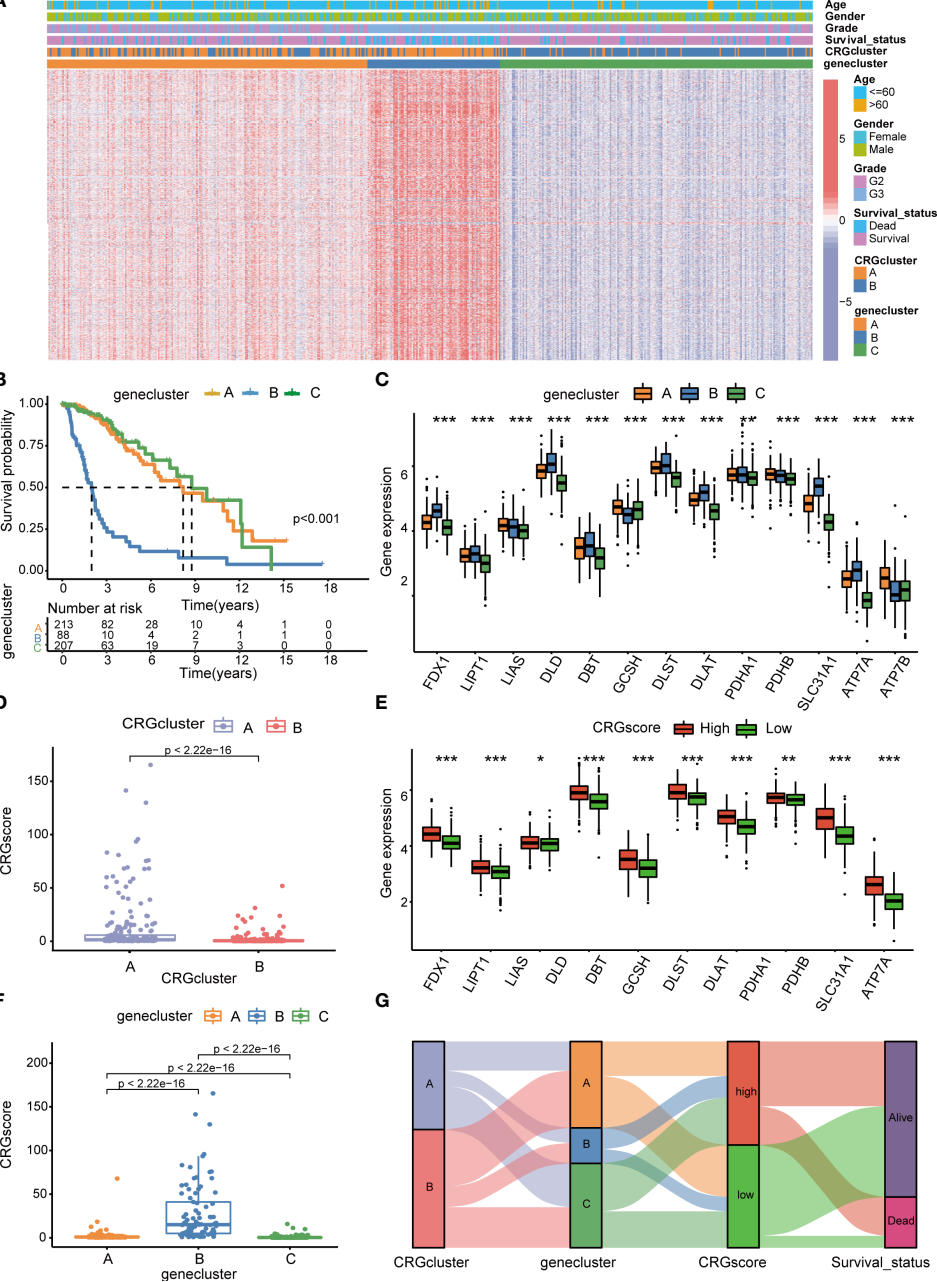
Identification of cuproptosis gene clusters and construction of the cuproptosis-related prognostic model in LGG. **(A)** The heatmap showed the different expressions of overall survival (OS)–DEGs and clinicopathological characteristics among gene cluster A to C **(B)** Kaplan–Meier OS curves for patients in the three gene clusters (log-rank test). **(C)** Differences in the expression levels of 13 CRGs among gene cluster A to C. **(D)** Differences in CRG scores between CRG cluster A and B **(E)** Differences in the expression levels of 13 CRGs between high- and low-risk groups. **(F)** Differences in CRG scores among gene cluster A to C, **(G)** Sankey diagram of subtype distributions in groups with different CRG scores and survival outcomes. *P*-values were shown as: *P< 0.05; **P< 0.01; ***P< 0.001. LGG, lower-grade glioma (WHO II and III); OS, overall survival; DEGs, differentially expressed genes; CRGs, cuproptosis-related genes.

Subsequently, the prognostic values of the above 1,966 CRG cluster-related DEGs were assessed by univariate Cox regression analysis and a total of 1,424 genes associated with OS (OS-related DEGs) were screened out (*P*< 0.05). Based on the expression levels of OS-related DEGs, a consensus clustering algorithm was employed and LGG patients were classified into three gene clusters, termed gene cluster A (n=215), gene cluster B (n=88), and gene cluster C (n=208) (**Supplementary Figures 4A**–**H**). PCA analysis revealed that gene clusters could be identified clearly (**Supplementary Figure 4I**). The expression profiles and clinical information of OS-DEGs in different gene clusters are shown in **Figure 5A**. We found that gene cluster B was correlated with high gene expression levels, an advanced grade (G3), and more death events. Patients in gene cluster B were proven to be related to worse prognosis than those in gene cluster A and C by Kaplan–Meier analysis (*P*< 0.001) (**Figure 5B**). Furthermore, CRGs were differentially expressed among three gene clusters, with the highest expression levels in gene cluster B and the lowest expression levels in gene cluster C. (**Figure 5C**).

### Construction and evaluation of the cuproptosis-related prognostic model

We performed LASSO and multivariate Cox regression analysis for 1,424 OS-related DEGs to establish the cuproptosis-related prognostic model. Patients in the TCGA cohort were divided into training and test cohorts at a ratio of 1:1. In the TCGA training cohort, 11 genes were obtained, followed by LASSO Cox regression analysis, and were subjected to multivariate Cox regression analysis (**Supplementary Figure 5**). Ultimately, five key genes remained, including Chromosome 21 Open Reading Frame 62 (*C21orf62*), Dorsal Inhibitory Axon Guidance Protein (*DRAXIN*), ITPR Interacting Domain Containing 2 (*ITPRID2*), Mitogen-Activated Protein Kinase Kinase Kinase 1 (*MAP3K1*) and Monooxygenase DBH Like 1 (*MOXD1*). Further GSEA analyses based on a single gene expression indicated that B-cell-mediated immunity, complement cascade, interferon-gamma response, chromosome segregation, mitotic spindle checkpoint, G2M checkpoint, T-cell receptor complex, CD22-mediated BCR regulation, epithelial–mesenchymal transition, cell cycle checkpoints, voltage-gated potassium channels, and other biological processes related to immunity or cancer were significantly enriched (**Supplementary Figure 6**). Next, we constructed a five-gene cuproptosis-related prognostic model and the associated CRG score can be calculated as follows: CRG_score= 0.164859* *C21orf62* + 0.293187* *DRAXIN* + 0.882099* *ITPRID2* + 0.625577* *MAP3K1* + 0.256801* *MOXD1*. We also explored the relationship between CRG clusters, gene clusters, and CRG scores. We found that CRG scores in CRG cluster A were significantly higher than that in CRG cluster B, and the expression of CRGs were upregulated in the high-risk group, which suggested that a high CRG score and the high expression of CRGs were associated with tumorigenesis and metastasis (**Figures 5D, E**). Meanwhile, the rank order of the CRG score in gene clusters was B > A > C (**Figure 5F**). The Sankey diagram showed subgroup distributions in groups with different CRG scores and survival outcomes (**Figure 5G**).

**Figure 6:**
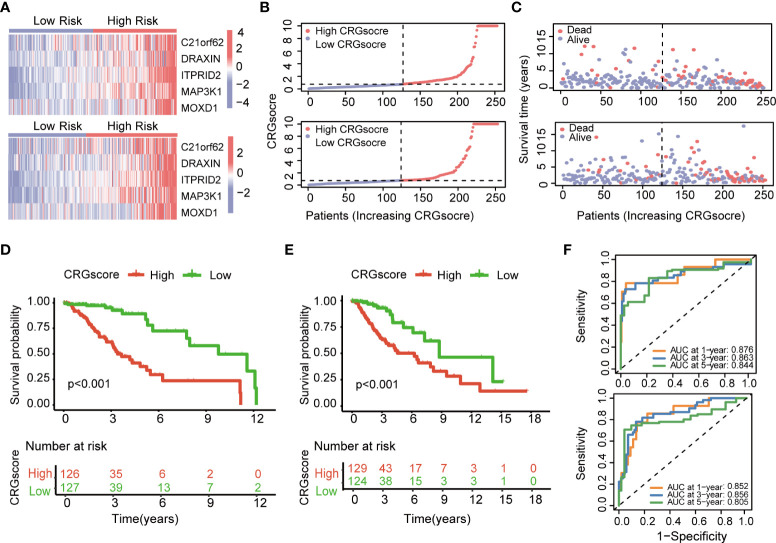
Evaluation of the cuproptosis-related prognostic model in the TCGA cohort. **(A)** The heatmap showed the different expressions of genes in the cuproptosis-related prognostic model between the high- and low-risk groups in TCGA training and test cohorts. **(B)** Distribution of the CRG score in TCGA training and testing cohorts. **(C)** The risk point plot showed the patterns of the survival time and survival status between the high- and low-risk groups in TCGA training and test cohorts. **(D)** The Kaplan–Meier OS curves for patients in the high- and low-risk groups in the TCGA training cohort (log-rank test). **(E)** The Kaplan–Meier OS curves for patients in the high- and low-risk groups in the TCGA testing cohort (log-rank test). **(F)** ROC curves showed the prognostic performance of the cuproptosis-related prognostic model in TCGA training and testing cohorts. TCGA, the Cancer Genome Atlas; CRGs, CRGs, cuproptosis-related genes; OS, overall survival.

The heatmap showed the different expressions of *C21orf62*, *DRAXIN*, *ITPRID2*, *MAP3K1*, and *MOXD1* between the high- and low-risk groups in TCGA training and test cohorts (**Figure 6A**). In addition, the risk plot of the CRG score revealed that patients with a high CRG score were related to more cases of death and shorter survival time (**Figures 6B, C**). Kaplan–Meier analysis showed that patients in the high-risk group had a worse OS than low-risk patients in both TCGA training and testing cohorts (*P*< 0.001) (**Figures 6D, E**). The 1-, 3-, and 5-year survival probability of the CRG score was represented by the AUC values of 0.876, 0.863, and 0.844, respectively, in the TCGA training cohort (**Figure 6F**). In the TCGA testing cohort, AUC values for predicting 1-, 3-, and 5-year OS were 0.852, 0.856, and 0.805. Meanwhile, the calibration curve also manifested a satisfactory agreement between predictive and observational values at the probabilities of 3- and 5-year survival (**Supplementary Figures 7A, B**).

### External validation of the cuproptosis-related prognostic model

**Figure 7:**
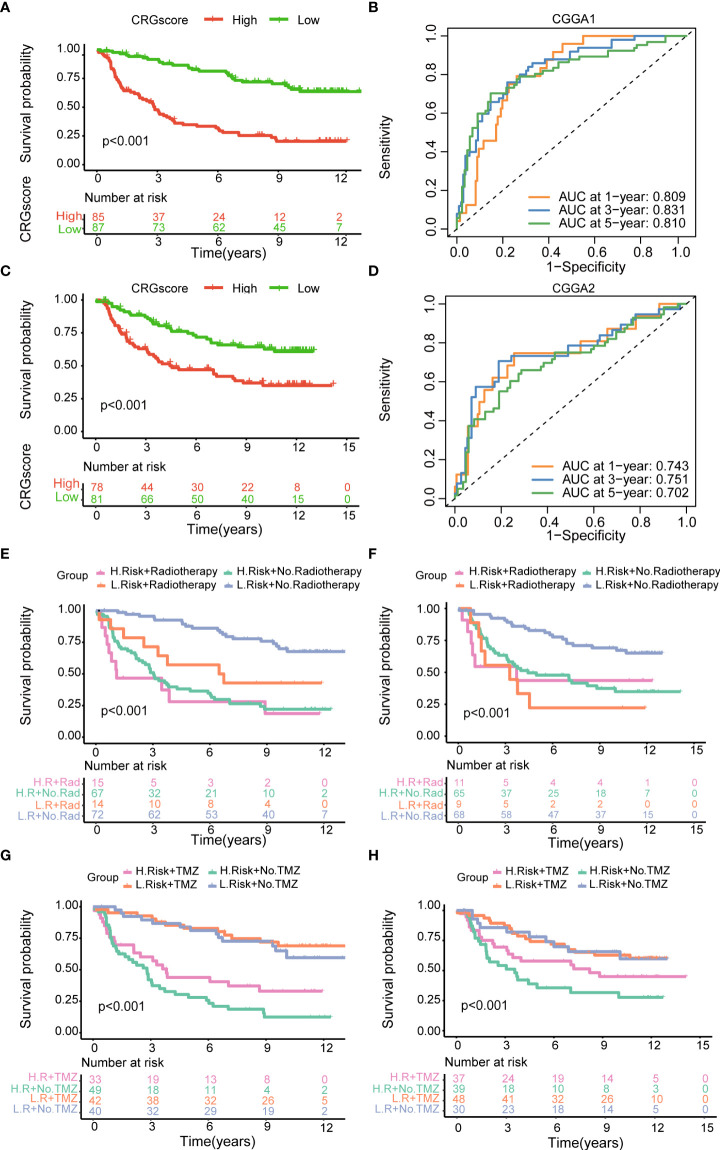
Validation of the cuproptosis-related prognostic model in CGGA cohorts. **(A)** The Kaplan–Meier OS curves for patients in the high- and low-risk groups in the CGGA1 cohort. **(B)** The Kaplan–Meier OS curves for patients in the high- and low-risk groups in the CGGA2 cohort. **(C)** ROC curves showed the prognostic performance of the cuproptosis-related prognostic model in the CGGA1 cohort. **(D)** ROC curves showed the prognostic performance of the cuproptosis-related prognostic model in the CGGA2 cohort. **(E–G)** The Kaplan–Meier OS curves among four groups classified by the CRG score and treatment with radiotherapy in CGGA1 and CGGA2 cohorts. **(G–H)** The Kaplan–Meier OS curves among four groups classified by the CRG score and treatment with TMZ in CGGA1 and CGGA2 cohorts. CGGA, Chinese Glioma Genome Atlas; ROC, receiver operating characteristic; OS, overall survival; TMZ, temozolomide; CRGs, cuproptosis-related genes.

To further verify the prognostic performance of the model, we applied two external validation cohorts (CGGA1 and CGGA2) (**Supplementary Table 7**). Patients were also classified into high- and low-risk groups according to CRG scores. Kaplan–Meier analysis showed a significantly better prognosis in the low-risk group compared to that in the high-risk group (**Figures 7A, B**). Meanwhile, the model also demonstrated a high AUC value in external validation cohorts (**Figure 7C, D**), and the calibration curve also exhibited a satisfactory agreement between predictive and observational values at the probabilities of 3- and 5-year survival (**Supplementary Figures 7C, D**).

Then, patients were stratified into four groups by the CRG score and treatment strategies. Kaplan–Meier analysis showed that patients in low-risk and no-radiotherapy groups had the best prognosis (**Figures 7E, F**). However, the predictive ability of the model was not impacted by TMZ treatment. Whether patients received TMZ or not, the low-risk group always showed a strong survival advantage (**Figures 7G, H**).

### Correlations of cuproptosis-related gene score with immune infiltration and cancer stem cell index in lower-grade glioma

**Figure 8:**
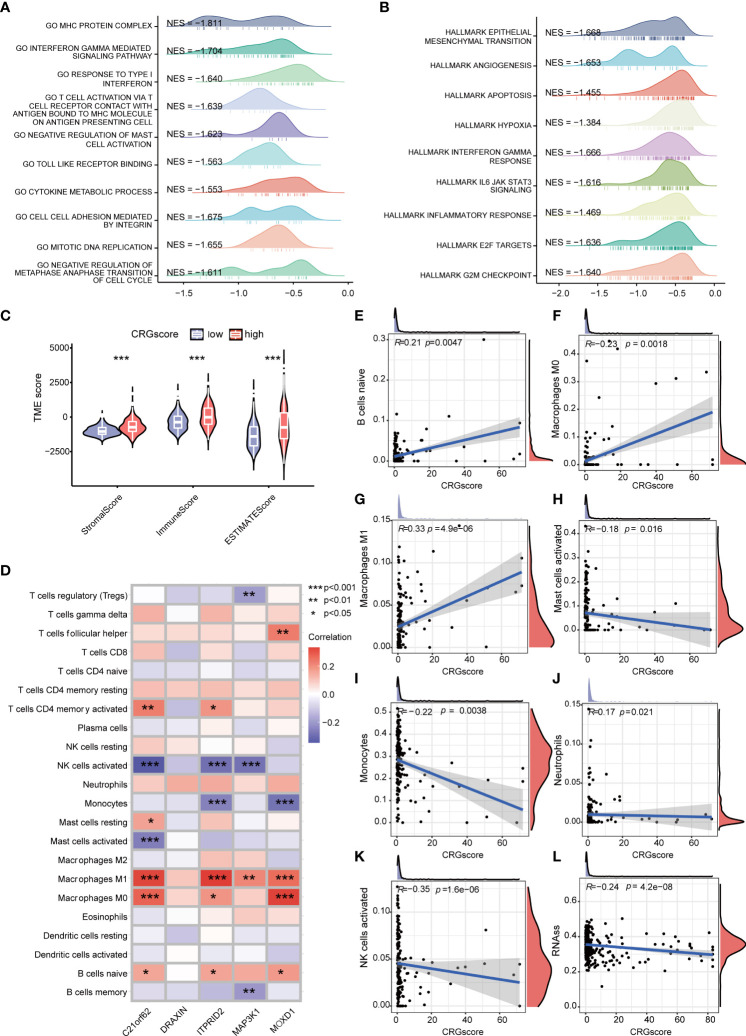
Correlations of the CRG score with immune infiltration and the cancer stem cell (CSC) index in LGG. **(A)** GSEA of significant GOBP terms enriched in the high-risk group. **(B)** GSEA of significant HALLMARK terms enriched in the high-risk group. **(C)** Correlations between CRG scores and TME scores. **(D)** Correlations between the abundance of immune cells and five genes in the cuproptosis-related prognostic model. **(E–K)** Correlations between the abundance of immune cells and the CRG score. **(L)** Correlations between the CSC index and the CRG score. *P*-values were shown as: *P< 0.05; **P< 0.01; ***P< 0.001. CRGs, cuproptosis-related genes; CSC, cancer stem cell; LGG, lower-grade glioma (WHO II and III); GSEA, gene set enrichment analysis; GOBP, gene ontology biological process; TME, tumor microenvironment.

GSEA was conducted to explore the potential biological functions between high- and low-risk groups (**Figures 8A, B**) (**Supplementary Table 8**). We observed that a high CRG score was mainly related to the cell cycle (negative regulation of metaphase–anaphase transition of the cell cycle, G2M checkpoint hallmark, E2F target hallmark), tumor progression (epithelial–mesenchymal transition hallmark, angiogenesis hallmark), and immunity (interferon gamma–mediated signaling pathway, T-cell activation *via* T-cell receptor contact with antigen bound to the MHC molecule on antigen-presenting cell, inflammatory response hallmark). TME scores including the stromal score, immune score, and ESTIMATE score were significantly higher in the high-risk group (**Figure 8C**). We assessed the correlation of immune infiltration with the five genes cuproptosis-related prognostic model (**Figure 8D**). Macrophage M0 and Macrophage M1 were positively correlated with the CRG score, while mast cells activated, monocytes, neutrophils, and NK cells activated were negatively correlated with the CRG score (**Figures 8E–K**).

Cancer stem cells are a group of cells with the features of self-renewing, being multipotent, and tumor-initiating, which can drive the growth and recurrence of tumors and are resistant to many current treatments. The CRG score and CSC index were synthesized to evaluate their relationship. A mild but significant negative correlation (*R* = -0.24, *P* = 4.2e-08) was detected (**Figure 8L**).

### Correlations of cuproptosis-related gene score with tumor mutational burden and immune checkpoint genes in lower grade glioma

**Figure 9:**
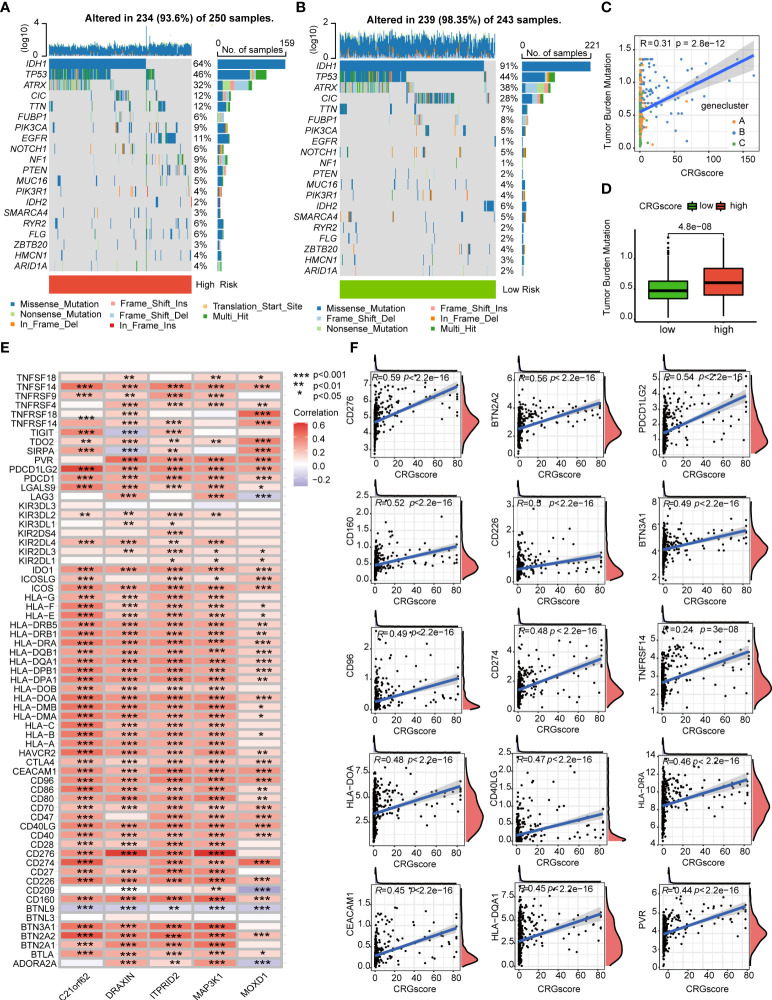
Correlations of the CRG score with TMB and ICGs in LGG. **(A, B)** The mutational landscape of LGG patients in high- and low-risk groups. **(C)** Correlations between the TMB and the CRG score in different gene clusters. **(D)** Difference in the TMB scores between high- and low-risk groups. **(E)** Correlations between the expression of ICGs and five genes in the cuproptosis-related prognostic model. **(F)** Correlations between the expression of ICGs and the CRG score. *P*-values were shown as: *P< 0.05; **P< 0.01; ***P< 0.001. CRGs, cuproptosis-related genes; TMB, tumor mutation burden; ICGs, immune checkpoint genes; LGG, lower-grade glioma (WHO II and III).

Previous studies demonstrated that high TMB scores are associated with increased treatment response to immunotherapy. Here, patients in the high-risk group (64%) had a markedly lower mutation incidence of *IDH1* than those patients in the low-risk group (91%) (**Figures 9A, B**). The TMB score was significantly higher in the high-risk group (*P* = 4.8e-0.8), and the CRG score was positively correlated with the TMB score (*R* = 0.31, *P* = 2.8e-12) (**Figures 9C, D**). Considering that the expression levels of ICGs have been reported to correlate with the clinical benefit of checkpoint blockade immunotherapy (35, 36), we further explored the relationship between the CRG score and ICGs. We found that most ICGs were significantly correlated with the five genes in the model (**Figure 9E**). The expression levels of 61 ICGs including *CD276*, *BTN2A2*, *PDCD1LG2*, *CD274*, and *CD40LG* increased with the increasing CRG score (**Figure 9F**). Only *BTNL9* was negatively correlated with the CRG score (*R* = -0.24, *P* = 2.6e−08) (**Supplementary Table 9**). Then, we found that patients with a high CRG score and high expression levels of *CD276*, *CD274*, *BTN2A2*, *PDCD1LG2*, and *CD40LG* were associated with poor OS than others (**Supplementary Figure 8**).

### Estimation of cuproptosis-related prognostic model in immunotherapy response

**Figure 10:**
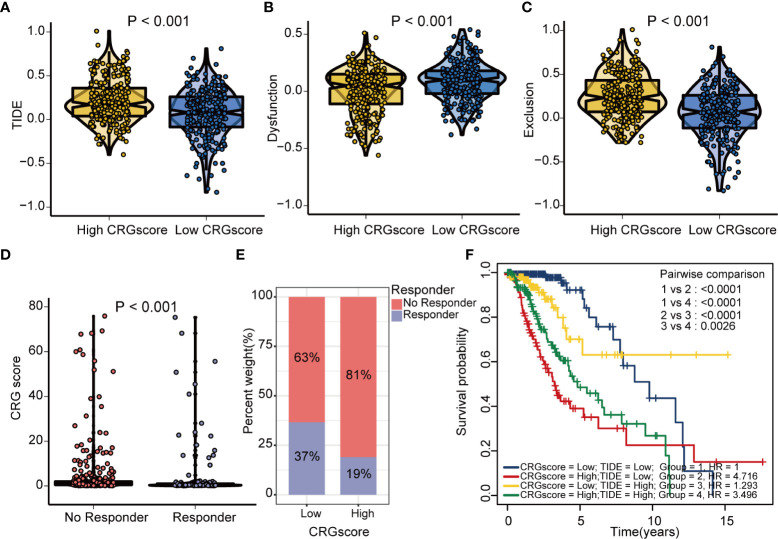
Estimation of the cuproptosis-related prognostic model in immunotherapy response in LGG. **(A)** Difference in TIDE scores between high- and low-risk groups. **(B)** Difference in dysfunction scores between high- and low-risk groups. **(C)** Difference in exclusion scores between high- and low-risk groups. **(D)** Difference in CRG scores between responder and no responder groups based on the TIDE algorithm. **(E)** The distribution of immunotherapy response in indicated groups stratified by the CRG scores based on the TIDE algorithm. **(F)** The Kaplan–Meier OS curves among four groups classified by the CRG score and TIDE score. TIDE, tumor immune dysfunction and exclusion; CRG, cuproptosis-related genes; OS, overall survival.

**Figure 11:**
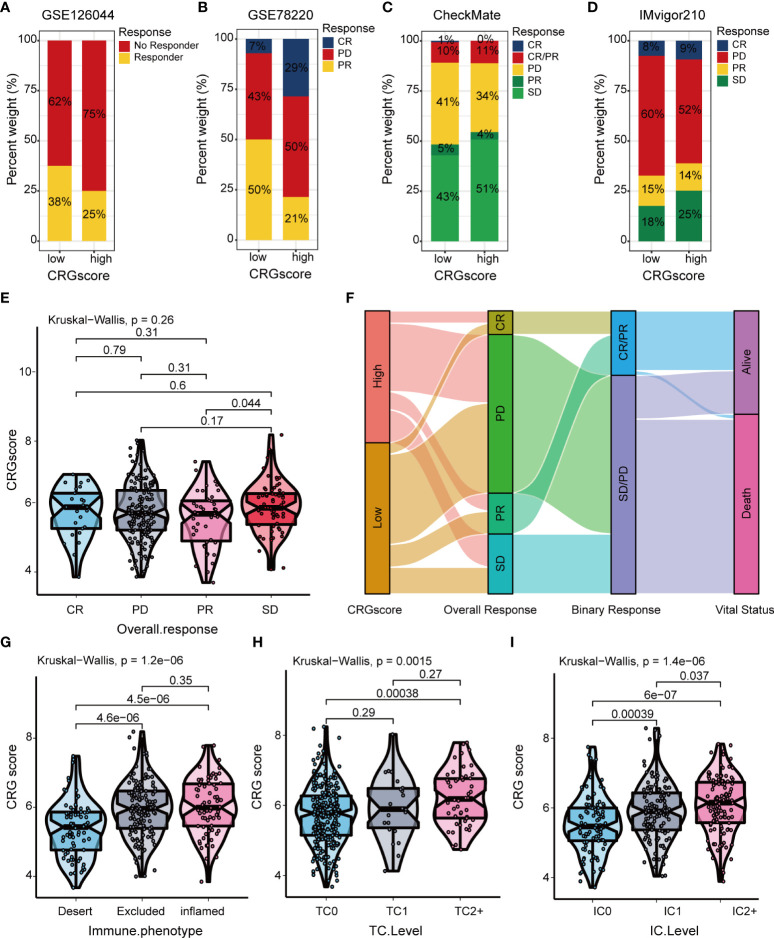
Correlation of the CRG score with immunotherapy response in mutiple cohorts. The distribution of immunotherapy response in indicated groups stratified by CRG scores in **(A)** GSE126044, **(B)** GSE78220, **(C)** the CheckMate cohort, and **(D)** the IMvigor210 cohort. **(E)** Difference in CRG scores among four immunotherapy response groups in the IMvigor210 cohort. **(F)** Sankey diagram of subtype distributions in groups with different CRG scores and immunotherapy response in the IMvigor210 cohort. Differences in the CRG score among **(G)** three immune phenotypes, **(H)** three TC levels, and **(I)** three immune cell (IC) levels in the IMvigor210 cohort. CRGs, cuproptosis-related genes; TC, tumor cell; IC, immune cell.

The TIDE algorithm was applied to estimated immunotherapy response for LGG patients based on the transcriptomic data. The results showed that the TIDE score of the high-risk group was significantly higher than that of the low-risk group, indicating that the patients in the low-risk group could benefit more from immunotherapy (**Figure 10A**). Patients in the low-risk group were associated with a higher dysfunction score and lower exclusion score (**Figures 10B, C**). Moreover, patients were classified into no responders and responders by the TIDE algorithm. We found that immunotherapy responders were correlated with a lower CRG score (**Figures 10D, E**). Patients with a combination of a high CRG score and high TIDE score group showed an association with the worst prognosis (**Figure 10F**). Then, we validated the predictive value of the cuproptosis-related prognostic model in immunotherapy response. The clinical response to immunotherapy in patients with non-small cell lung cancer or metastatic melanoma in the low-risk group compared to those in the high-risk group were confirmed (**Figures 11A, B**). However, no differences were identified in renal cell carcinoma patients and urothelial cell carcinoma patients (**Figures 11C–E**). **Figure 11F** shows the subtype distributions in groups with different CRG scores and immunotherapy response in the IMvigor210 cohort. Furthermore, in the IMvigor210 cohort, the CRG scores were different among different immune phenotypes, tumor cell (TC) levels, and immune cell (IC) levels (**Figures 11G–I**).

### Correlations of cuproptosis-related gene score with chemoradiotherapy sensitivity–related genes and chemotherapeutic sensitivity in lower-grade glioma

**Figure 12:**
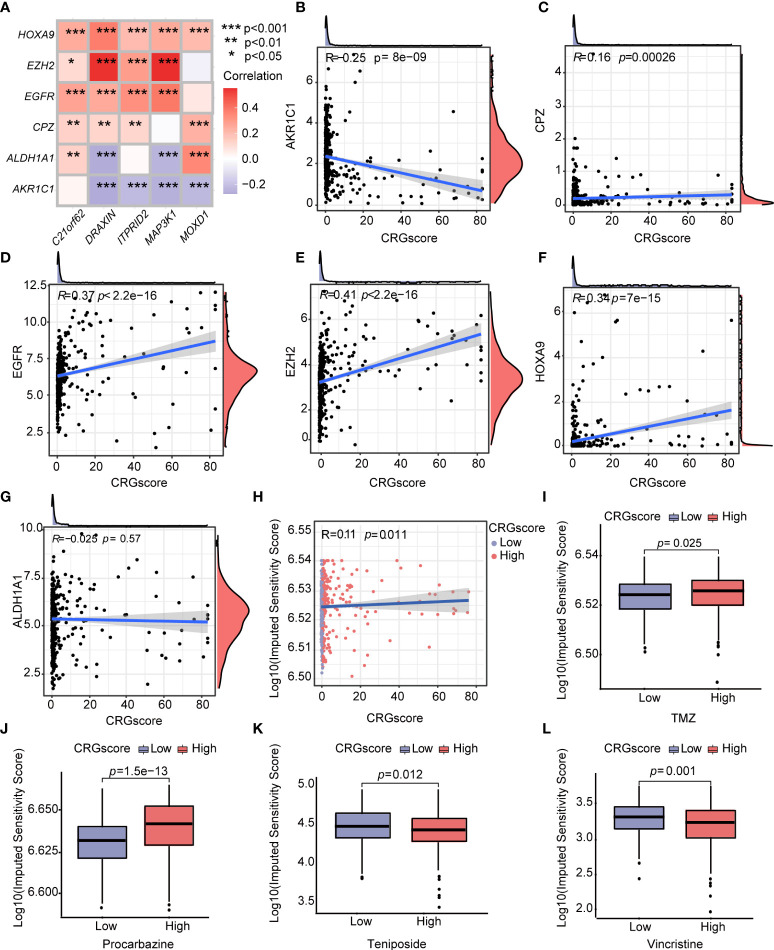
Correlations of the CRG score with CRSGs and chemotherapeutic sensitivity in LGG. **(A)** Correlations between the expression of CRSGs and five genes in the cuproptosis-related prognostic model. **(B–G)** Correlations between the expression of CRSGs and the CRG score. **(H)** Correlations between the imputed sensitivity score of TMZ and the CRG score. **(I–L)** Difference in chemotherapeutic sensitivity between high- and low-risk groups. *P*-values were shown as: *P< 0.05; **P< 0.01; ***P< 0.001. CRSGs, chemoradiotherapy sensitivity–related genes; CRGs, cuproptosis-related genes; TMZ, temozolomide.

Additionally, we observed a strong correlation between the expression levels of CRSGs and the expression levels of *C21orf62*, *DRAXIN*, *ITPRID2*, *MAP3K1*, and *MOXD1* (**Figure 12A**). As the CRG score increased, the expression levels of *CPZ*, *EGFR*, *EZH2*, and *HOXA9* increased, but the expression levels of *AKR1C1* decreased, which revealed a potential association between the CRG score and chemoradiotherapy (**Figures 12B–G**). To explore the values of the CRG score as a biomarker to predict the chemotherapeutic response in LGG patients, we performed drug sensitivity analysis using the “oncoPredict” R package. A higher imputed sensitivity score represented lower sensitivity to the drug. We found patients in the high-risk group had higher imputed sensitivity scores of TMZ and procarbazine, while the imputed sensitivity scores of teniposide and vincristine were lower in patients in the high-risk group (**Figures 12H–L**).

### Independent prognostic analysis and establishment of a nomogram

**Figure 13:**
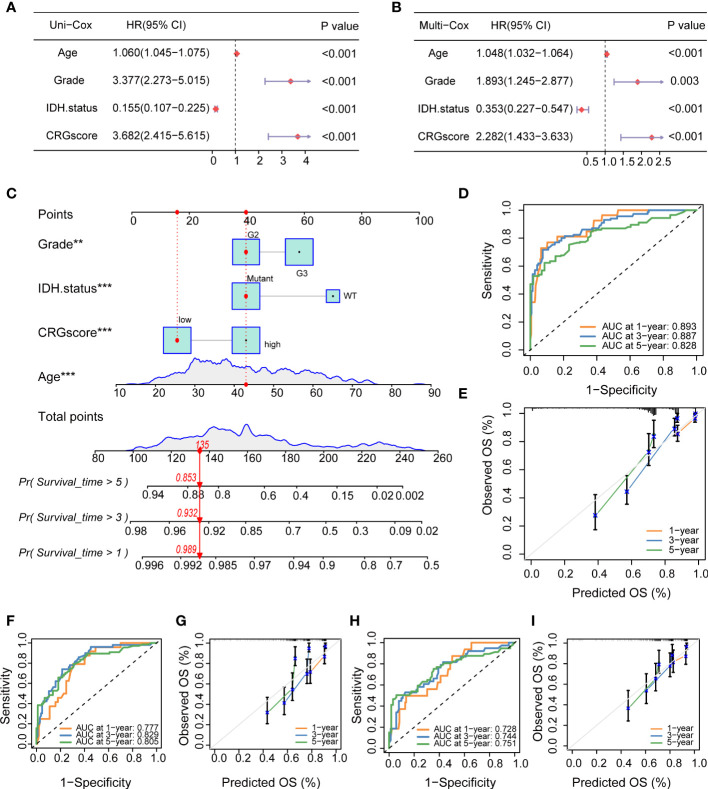
Independent prognostic analysis and establishment of a nomogram. **(A)** Univariate Cox regression analysis of the CRG score and clinical characteristics in the TCGA cohort. **(B)** Multivariate Cox regression analysis of the CRG score and clinical characteristics in the TCGA cohort. **(C)** The nomogram was extablished to predict 1-, 3-, and 5-year overall survival probability of LGG patients in the TCGA cohort. **(D)** ROC curves showed the prognostic performance of the model in the TCGA cohort. **(E)** The calibration curves measured the relationship between the outcomes predicted by the model and the observed outcomes in the TCGA cohort. **(F)** ROC curves showed the prognostic performance of the model in the CGGA1 cohort. **(G)** The calibration curves measured the relationship between the outcomes predicted by the model and the observed outcomes in the CGGA1 cohort. **(H)** ROC curves showed the prognostic performance of the model in the CGGA2 cohort. **(I)** The calibration curves measured the relationship between the outcomes predicted by the model and the observed outcomes in the CGGA2 cohort. CRGs, cuproptosis-related genes; TCGA, the Cancer Genome Atlas; LGG, lower-grade glioma (WHO II and III); ROC, receiver operating characteristic; CGGA, Chinese Glioma Genome Atlas.

To determine if the CRG score could be used as an independent prognostic predictor for OS, we combined clinical characteristics and the CRG score to conduct univariate and multivariate Cox regression analyses. In the TCGA cohort, age, grade, IDH mutation status, and CRG score demonstrated significant differences (**Figures 13A, B**). The prognostic value of the CRG score was also verified in external CGGA cohorts, and the results showed that CRG score is an independent prognostic predictor for LGG patients (**Supplementary Figure 9**).

Then, we combined the CRG score and clinical chrematistics (age, grade, IDH mutation status) to establish a nomogram in the TCGA cohort, which exhibited a quantitative method to generate personalized predictions for LGG patients (**Figure 13C**). The AUC values of the model were estimated, and calibration analyses were performed to assess the predictive ability and accuracy for prognosis. **Figure 13D** showed that 1-, 3- and 5-year AUC values of the model in the TCGA cohort were 0.893, 0.887, and 0.828, respectively. The model also had good predictive ability in the CGGA1 cohort (AUC > 0.75) and CGGA2 cohort (AUC > 0.7) (**Figures 13F, H**). Subsequently, the calibration plots demonstrated good agreement between model-predicted probability and the observed outcomes (**Figures 13E, G, I**).

### Tissue samples, quantitative real-time PCR, and Western blotting

**Figure 14:**
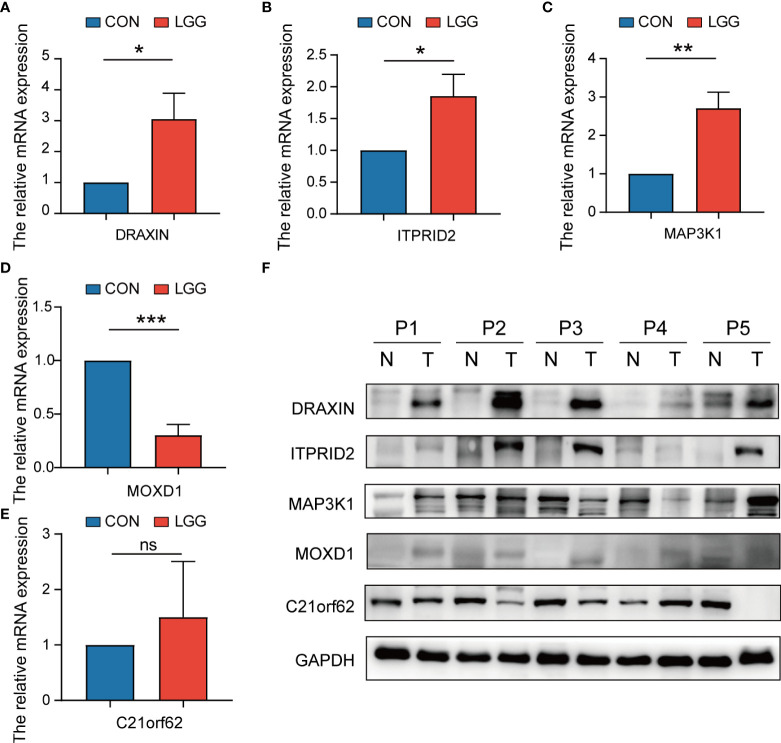
The expressions of five signature genes were validated by quantitative real-time PCR (qRT-PCR) and Western blotting (WB). **(A–E)** Expression of genes at the mRNA level by qRT-PCR. **(F)** Expression of genes at the protein level by WB. qRT-PCR, quantitative real-time PCR; WB, Western blotting. *P< 0.05; **P< 0.01; ***P< 0.001. ns, no significance.

To verify the expression level of signature genes in LGG, we collected five paired cancer- and adjacent normal tissues from SYSUCC. As shown in **Figures 14A–D**, qRT-PCR showed that the expression of the *DRAXIN*, *ITPRID2*, and *MAP3K1* were significantly upregulated while *MOXD1* was downregulated in tumor samples. Nevertheless, there was no significant difference in the expression of *C21orf62* (**Figure 14E**). Further investigation indicated heterogeneity in the expression of *C21orf62*. Briefly, it was upregulated in two patients but downregulated in the remaining patients (the relative expression was 5.33, 1.68, 0.36, 0.07, and 0.04, respectively). The results of WB also demonstrated the heterogeneity in *C21orf62*, *MAP3K1*, and *MOXD1* but consistently a significant upregulation in *DRAXIN* and *ITPRID2* at the protein level (**Figure 14F**).

## Discussion

Cell death is critical for maintaining organismal homeostasis, developing, and preventing excessively proliferative malignancy (37, 38). As a hallmark of cancer, metabolism plays an indispensable role in cell death (39). Numerous studies have demonstrated a correlation between metabolism and multiple cell deaths in cancer (40). In gliomas, stress-induced cell death signaling usually involves the mitochondria and endoplasmic reticulum, which activates reactive oxygen intermediates and regulates lipid mediators in cell proliferation, migration, and interaction with endothelial and microglial cells (41, 42). Nevertheless, the underlying mechanisms and correlations among metabolism, mitochondria, and cell death in gliomas still remain poorly understood.

Cuproptosis, first named by Todd R. Golub, sheds light on copper metabolism and mitochondrial dysregulation in cell death. Several studies have focused on a single cuproptosis regulator in cancer; however, the overall effect of multiple CRGs has not been fully characterized. The specific role of cuproptosis in LGG remains unclear. Furthermore, although many prognostic signatures have been established based on the patterns of pseudogenes (43), N6-methylandenosine (44), immunity (45), ferroptosis (46), and pyroptosis (47) their predictive ability needs to be further improved, and there is still a lack of a prognostic signature based on the characteristics of cuproptosis. In the present study, we systematically investigated the global alterations in 13 CRGs at genetic and transcriptional levels in LGG, established a scoring system consisting of five signature core genes, and eventually constructed a quantitative nomogram by integrating the CRG score and clinicopathological features including grades, IDH status, and age. Compared to other models, our model has better performance overall and has better clinical application value (**Supplementary Table 10**).

Copper metabolism plays a crucial role in diverse biological processes (48). We first characterized the landscape of genetic and transcriptional variations of CRGs in LGG. Although only seven samples had genetic mutations in CRGs, we were surprised to find that 12 CRGs were upregulated between the LGG and normal tissues, while *ATP7B* was downregulated. We noticed that the expression of most CRGs was significantly correlated with poor prognosis. Based on CRG expression profiles, we further divided LGG patients into two distinct molecular subtypes with the unsupervised clustering approach. Compared to patients with subtype B, patients with subtype A had a worse prognosis within the early 9 years and more advanced clinicopathological features. We speculate that the differences may be partially attributed to different responses to treatment. To further validate the speculations, we analyzed the patterns of immune checkpoints and chemoradiotherapy-associated genes. *AKR1C1, EGFR, EZH2, HOX9, HGMT, SOX2*, and *TBX5* have been implicated in chemoradiotherapy resistance due to their roles in DNA damage repairing, signaling, angiogenesis, and TME remodeling (49–51). For example, the overexpression of *AKR1C1* can reduce the production of reactive oxygen species, eliminate free radicals, and inactivate anthracycline anticancer drugs, thereby decreasing DNA damage and inhibiting cell apoptosis, which can finally reduce the sensitivity of chemotherapy (24). High *EGFR* expression was associated with poor response to radiation or chemoradiotherapy, and specifically targeting EGFR and EGFR variant receptors is undergoing clinical evaluation in patients with glioma (52). Consistent with the above assumption, we verified that different subtypes vary significantly in the expression of the therapy-associated genes, including CRSGs and ICGs. In addition, the characteristics of the TME like the stromal score, immune score, and ESTIMATE score also indicated significant differences. Increasing evidence has shown that multiple immune cells play a vital role in the immune defense of LGG (53). The densities of tumor-infiltrating T cells were also proven to be critical for the initial stage and development of LGG, and the γδT cell can effectively recognize and kill tumor cells (54). Here, we observed striking differences in the type of CD4+ T cell, CD56+ T cell, γδT cell, and T helper2 cell between subtypes, which demonstrates a close relevance between cuproptosis and tumor immunity. These results strongly implied the potential roles of cuproptosis in LGG prognosis.

The essence of cuproptosis is a copper-induced cell death mediated by protein lipoylation (6). TCA enzymes (in particular, the PDH complex) are indispensable for initiating lipoylation, which contributes to the induction of HSP70 and is reflective of acute proteotoxic stress (7). Consistent with the facts, functional enrichment analyses demonstrated that protein alteration like localization to the microtubule organizing center, polyubiquitination, lysine degradation, ubiquitin-mediated proteolysis, and citrate cycle TCA cycle processes showed a significant difference in the subtypes we identified. Moreover, our results revealed that the G2M checkpoint, E2F targets, MYC targets, epithelial–mesenchymal transition, and angiogenesis were also mainly associated with different subtypes based on the CRG expression, which have been known in the progression of malignancies (55–57). In addition, the GO and KEGG results about the differential expression genes between subtypes indicated a close link between cuproptosis and the cell cycle as well as genomic stability, including organelle fission, nuclear division, and chromosome segregation. As many studies have illustrated the role of copper metabolism in cancer, our study added to the evidence that directly or indirectly targeting cuproptosis may bring a satisfactory effect in anti-glioma therapy.

Increasing evidence has illustrated the function of copper in the initial stage and progression of tumors at the transcriptomic level (58). In this study, mRNA transcriptome differences between distinct cuproptosis patterns have also been explored. Through multivariate Cox analyses about DEGs between CRG clusters, 1,424 OS-associated DEGs remained. Similar to the clustering of the CRG phenotypes, three genomic subtypes were identified based on the above DEGs, which demonstrated a close relationship with patients’ prognosis, indicating its predictive ability for LGG. To better evaluate the cuproptosis pattern of individual patients with LGG, we further constructed a predictive cuproptosis-related prognostic model and a CRG score system, which consisted of *C21orf62*, *DRAXIN*, *ITPRID2*, *MAP3K1*, and *MOXD1*. As a coding gene for chromosome 21 open reading frame, *C21orf62* has a great impact on the gene structure, thus regulating the biological homeostasis (59). *DRAXIN*, a recently identified axon guidance protein, is crucial for the formation of forebrain commissures and repulsion of netrin-stimulated spinal commissural axons (60, 61). *DRAXIN* also plays a vital role in lung carcinomas (62). However, its role in LGG remains elusive. *ITPRID2*, also known as *SSFA2*, has been reported in the development of many malignancies (63, 64). In LGG, the inhibition of *ITPRID2* can regulate the cell cycle to significantly reduce the proliferation ability and induce the early apoptosis rate (65). As a member of mitogen‐activated protein kinases, *MAP3K1* is an important regulator of evolutionarily conserved proteins in various cellular physiologies (66). Numerous studies have confirmed that the activation of MAPKs was positively correlated to the progression and therapy resistance of glioma (67). Based on the public database, Xie and his colleagues identified *MAP3K1* as a novel prognostic biomarker and potential therapeutic target in glioma (68). *MOXD1* has also been predicted to enable copper ion–binding activity (69), and *MOXD1* knockdown significantly suppresses the proliferation and tumor growth of glioblastoma cells *via* ER stress-inducing apoptosis (70). Accumulating evidence seems to indicate the potential roles of signature genes in glioma. In our validation experiments, although there was heterogeneity in their expressions at the protein level, almost all genes were significantly verified at the mRNA level. Further function enrichment analyses based on a single gene also demonstrated a close link between signature genes and immunity as well as malignant processes. Meanwhile, patients with gene cluster B exhibited a higher CRG score and the worst outcomes, and a higher CRG score is usually accompanied by higher expression levels of CRGs in LGG tissues. Correlation analysis among the CRG cluster, gene cluster, CRG score, and survival status further indicated our scoring system’s robust and stable prognostic-predictive ability. The distribution plots and K-M plot validated that survival times decreased when the CRG score increased in the TCGA training and validation cohorts. In addition, this prediction ability was further confirmed by two cohorts from CGGA as well. Furthermore, patients with low- and high-risk CRG scores showed significant differences in responses to radiotherapy or chemotherapy. Taken together, we demonstrated an independent and predictive role of CRG score in LGG.

Accumulative in-depth research has suggested some prognosis factors for LGG. Immunobiology has been acknowledged as a dominant factor for malignant processes (71). Additionally, the immune infiltrating cell signature has also been indicated as a prognostic marker in gliomas (72, 73). As copper has been strongly indicated in the regulation of immunity (74), identifying the role of cuproptosis in the TME cell infiltration might enhance our understanding of LGG antitumor therapy response, thus guiding more effective immunotherapy strategies. In this study, GSEA enrichment analysis illustrated the key role of immune processes between high- and low-risk CRG score patients. The patterns characterized by the immune-inflamed phenotype, higher stromal score, and higher ESTIMATE score exhibited a higher CRG score, while the other showed a lower CRG score. Five OS-associated gene signatures were found to be significantly correlated with immune cells. In addition, a higher CRG score was positively associated with the infiltration of B cells naive, macrophages M0 and M1, T cells CD4 memory activated and T cells follicular helper, while it was negatively correlated with mast cells activated, monocytes, and NK cells activated. Evidence has shown the crucial roles of these cells in LGG. Myeloid-derived suppressive cells were reported to promote B-cell-mediated immunosuppression *via* the transfer of PD-L1 in gliomas (75). Infiltrated tumor-associated macrophages have been revealed to be negatively associated with the survival of glioma patients, and its related prognostic model based on MScores demonstrated a high accuracy rate (76). T cells were thought to infiltrate gliomas at an early stage, mediating immunosuppression and resistance to treatment (77). NK-cell-targeted therapies have also been highlighted in the immune escape of IDHmut gliomas (78). As immune cells participate in various biologies, including tumorigenesis and progression of LGG, targeting these cells may benefit LGG patients with unfavorable prognosis. Meanwhile, combined with the potential correlation between the above signature genes and immune checkpoints, targeting these immune checkpoints may benefit patients more. Furthermore, we noticed that the RNA stemness score increased with increasing CRG score, suggesting the critical role of cuproptosis patterns in LGG tumor maintenance.

Despite great advances in LGG therapy over the past decade, a substantial room for progress remains and improvements are in demand. Even standard-of-care multimodal treatment approaches including surgery, radiation, immunotherapy, and chemotherapy have been proposed (79); LGG patients show heterogeneity in their treatment response and outcomes when considering the pathologic features, especially in terms of mutation and therapy resistance (80). Further studies are required to assess the impact of intratumor heterogeneity and its TMB characteristic on prognosis and response to treatment. Alireza Mansouri and his colleagues reported that the methylation of the *MGMT* promoter provides better outcome prediction when patients receive temozolomide chemotherapy (81). *IDH1/2* mutation, which induced a high concentration of 2-hydroxyglutarate through the upregulation of *HIF-1α* and *VEGF*, could also benefit the patients (82). Similar to previous studies, our data revealed a markable difference in tumor mutation burden between CRG score subgroups. Patients with a low CRG score showed a higher rate of *IDH1* and *IDH2* mutation. In addition, the lower mutation rates of *TP53*, *EGFR*, and *PTEN* were also observed in low-risk patients, which has been demonstrated to correlate with worse clinical outcomes (83). Additionally, it has been reported that *ATRX* and *CIC* can also influence the prognosis. The profiling of gliomas has revealed that the majority (~75%) of low-grade gliomas that carry *IDH1* and *TP53* mutations also harbor *ATRX* mutations, thus underscoring their crucial role in gliomagenesis, which has been indicated in the procession of impairing non-homologous end-joining DNA repair (84, 85). Recent genomic analyses of brain cancers have implicated *CIC* as a critical suppressor gene in diffuse gliomas (86). Here, we discovered the significant mutations of these genes in our cohort, which provides a deeper understanding of LGG. Given the correlation between TMB and enhanced clinical response to immunotherapy, we further explored immunotherapy effectiveness in the subgroups of LGG. In the present study, 33 immune checkpoints were observed to be differentially expressed in the two groups. Five CRG signatures and the total CRG score were respectively associated with *CD276*, *BTN2A2*, and *PDCD1LG2*, which may be a potential treatment response predictor in the clinic.

Furthermore, we explored the role of cuproptosis patterns in the radiation and chemotherapy of LGG. Results showed a correlation between the CRG score and expression profiles of radiotherapy-associated genes. Targeting CRGs might contribute to enhancing therapeutic effects. Moreover, it has been considered reasonable to apply adjuvant temozolomide for patients with gliomas (87). TMZ can act as a radiosensitizer and be given full consideration to be part of standard treatment for newly diagnosed glioblastoma (88, 89). In our study, we discovered that patients with low CRG scores were more susceptive to the TMZ and procarbazine, suggesting a higher response to treatment and better clinical outcomes. Though low-risk patients in CGGA here did not benefit from the addition of TMZ, the complexity of TMZ in LGG is still worth exploring. Finally, to further improve the performance and facilitate the application of the CRG score, we established a quantitative nomogram that can be used for the prognosis stratification of LGG patients. Given the superior performance validated by multiple cohorts, the nomogram enables patients and physicians to create a more individualized surveillance program for LGG, thus improving the prognosis.

Nevertheless, there are still several issues to be addressed. First, the CRG risk signature was conducted based on the data retrospectively obtained from public databases, which was inevitably limited by an inherent case selection bias. More large-scale prospective and multicenter clinical studies are needed to confirm our findings. Second, some critical clinical variables like chemoradiotherapy and surgery were lacking for analysis in some datasets, which may influence the results of treatment response and cuproptosis state analyses. Though we analyzed several immunotherapy datasets in this study, most of them were not based on data from LGGs, which should be further studied. Furthermore, more clinical pathology samples should be included for validating the expression of signature genes, and more functional *in vivo* or *in vitro* experiments are further needed to verify the roles of signature genes in the future.

## Conclusion

Briefly, we demonstrated a comprehensive overview of CRG profiles in LGG and established a novel risk model for LGG patients’ therapies status and prognosis, which was partially constituted by a 5-CRG signature (*C21orf62*, *DRAXIN*, *ITPRID2*, *MAP3K1* and *MOXD1*). We also determined the roles of these genes in LGG by affecting the tumor-immune-stromal microenvironment, clinical features, therapy strategies, and prognosis. These findings highlight the potential clinical implications of CRGs, suggesting that cuproptosis may be the potential therapeutic target for patients with LGG.

## Data availability statement

The analyzed data could be obtained at TGCA (https://portal.gdc.cancer.gov/) and CGGA (www.cgga.org.cn) databases. The code applied in the study is available from the corresponding author on reasonable request. The accession number(s) can be found in the article/**Supplementary Material**.

## Ethics statement

The studies involving human participants were reviewed and approved by The Institutional Ethics Committee for Clinical Research and Animal Trials of the Sun Yat-sen University Cancer Center (B2022-246-01). The patients/participants provided their written informed consent to participate in this study.

## Author contributions

JB developed the study concept and design, JB and WC-L performed data acquisition, bioinformatics and statistical data analyses. JB, WC-L and HD drafted the manuscript together. HD collected clinic samples. WC-L and YY performed the validation experiments. JB, WC-L and HD constructed the figures and tables. WT-L, YL and YS were responsible for the integrity of the entire study and manuscript review. All authors contributed to the article and approved the submitted version.

## Funding

This study was supported by the National Natural Science Foundation of China (81800996).

## Conflict of interest

The authors declare that the research was conducted in the absence of any commercial or financial relationships that could be construed as a potential conflict of interest.

The reviewers RS and FD declared a shared affiliation with the authors to the handling editor at time of review.

## Publisher’s note

All claims expressed in this article are solely those of the authors and do not necessarily represent those of their affiliated organizations, or those of the publisher, the editors and the reviewers. Any product that may be evaluated in this article, or claim that may be made by its manufacturer, is not guaranteed or endorsed by the publisher.
