# Supplementary material for: Identification of a novel cuproptosis-related gene signature and integrative analyses in patients with lower-grade gliomas
Source: Front Immunol. 2022 Aug 15;13:933973. doi: 10.3389/fimmu.2022.933973 (PMC9420977; doi:10.3389/fimmu.2022.933973)
Supplement: Supplementary file 1 [file DataSheet_1.docx]

Supplementary Material

# Supplementary Figures and Tables

**
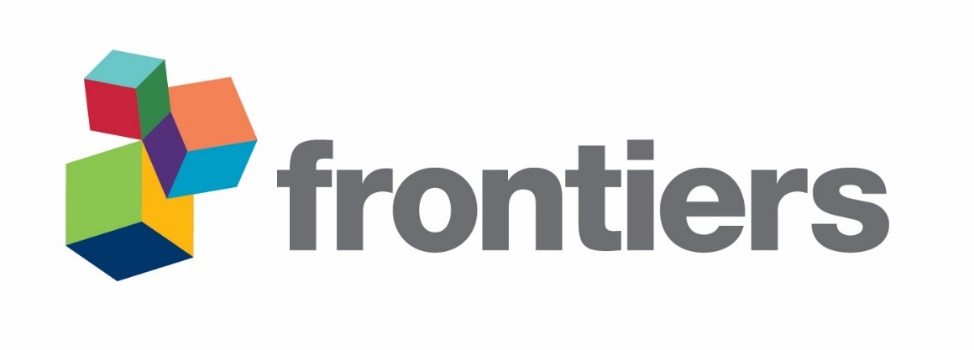
**

## Supplementary Figures


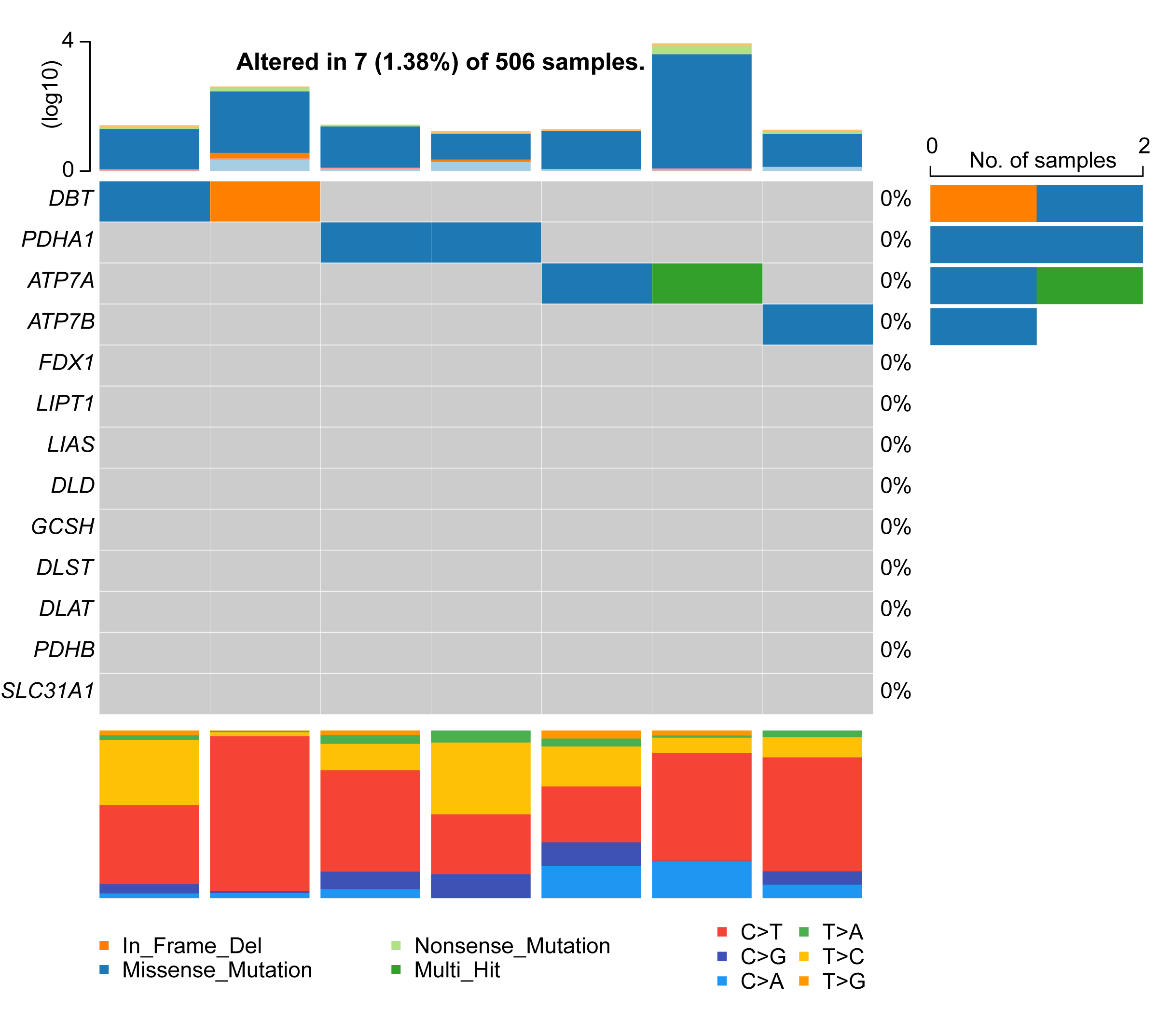


**Supplementary Figure 1.** Landscape of genetic variations of 13 CRGs in TCGA cohort. CRGs, cuproptosis related genes; TCGA, the Cancer Genome Atlas.


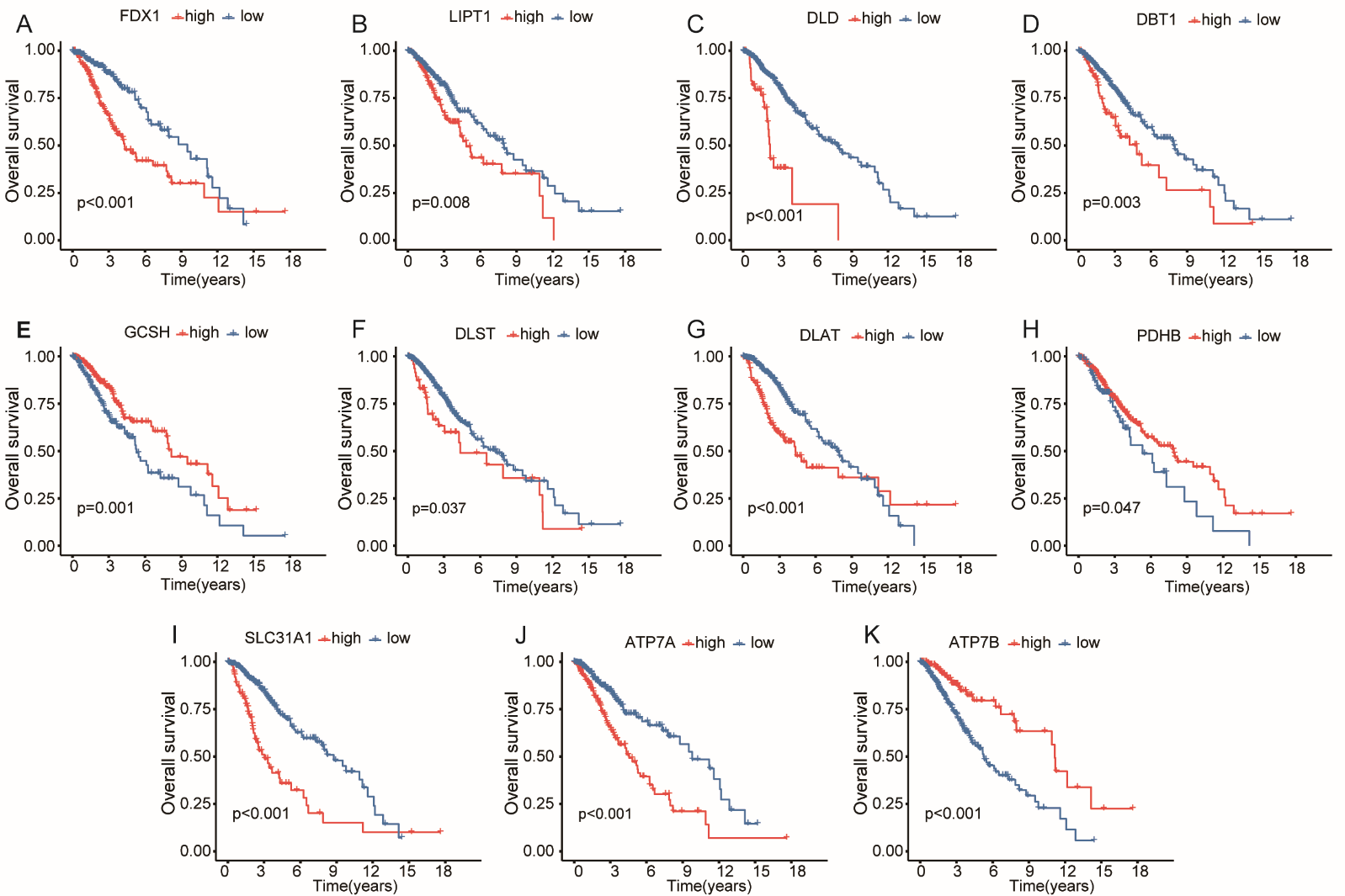


Supplementary Figure 2. Kaplan-Meier analysis of CRGs in TCGA cohort. Kaplan–Meier curves of overall survival between high and low expression levels of (A) *FDX1,* (B) *LIPT1*, (C) *DLD*, (D) *DBT1*, (E) *GCSH*, (F) *DLST*, (G) *DLAT*, (H) *PDHB*, (I) *SLC31A1*, (J) *ATP7A* and (K) *ATP7B* in TCGA-LGG cohort. CRGs, cuproptosis related genes; TCGA, the Cancer Genome Atlas; LGG, lower-grade glioma (WHO Ⅱ and Ⅲ).


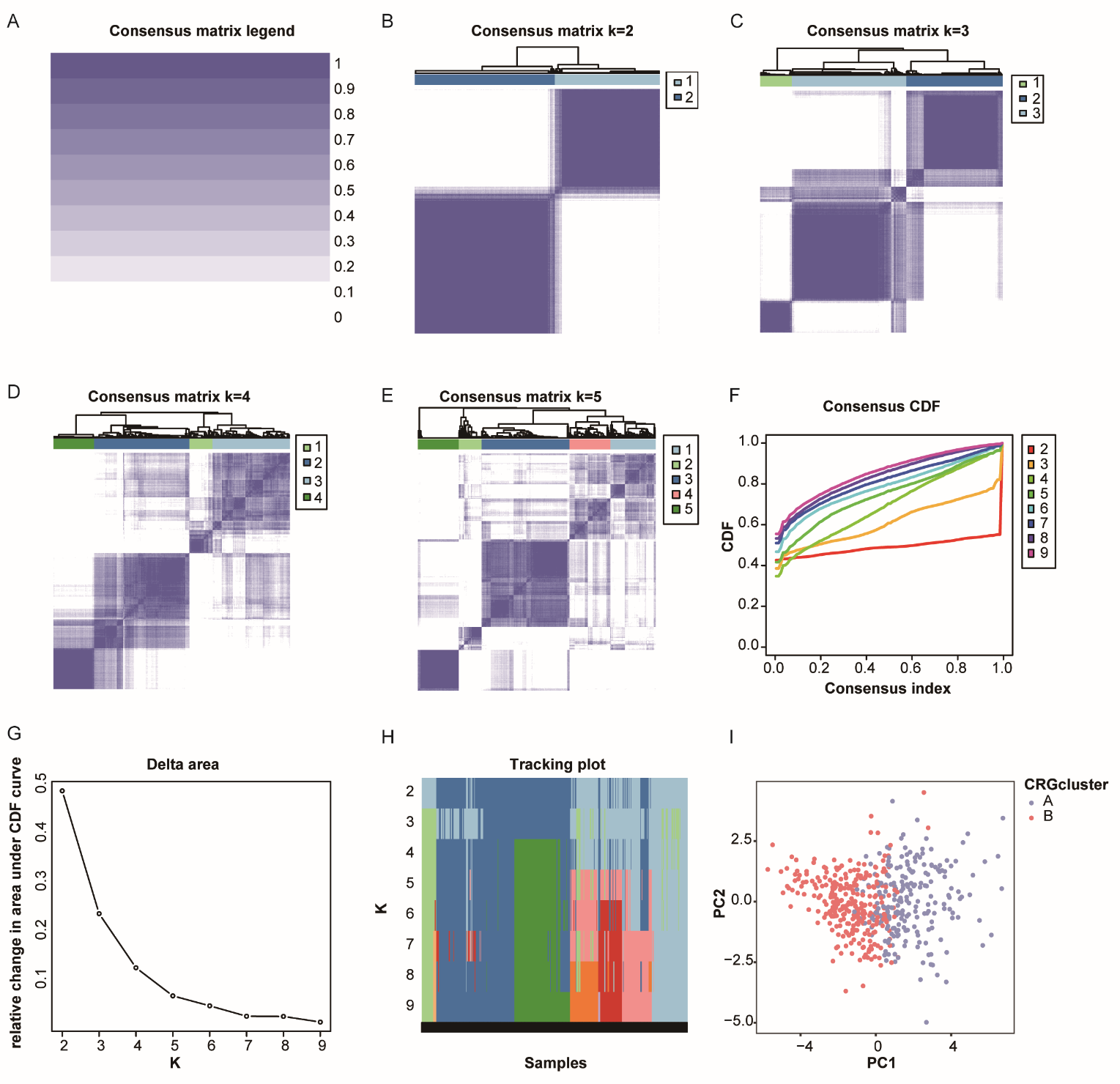


Supplementary Figure 3. Unsupervised clustering for CRGs. (A-E) TCGA-LGG cohort was grouped into 2 clusters according to the consensus clustering matrix (k = 2). (F) Uniform clustering CDF with k from 2 to 9. (G) The change of area under CDF curve with k from 2 to 9. (H) The tracking plot showed the relationship between samples and clusters. (I) The PCA plot showed the distribution of samples among 2 CRG clusters. CRGs, cuproptosis related genes; TCGA, the Cancer Genome Atlas; LGG, lower-grade glioma (WHO Ⅱ and Ⅲ); CDF, cumulative distribution function; PCA, principal component analysis.


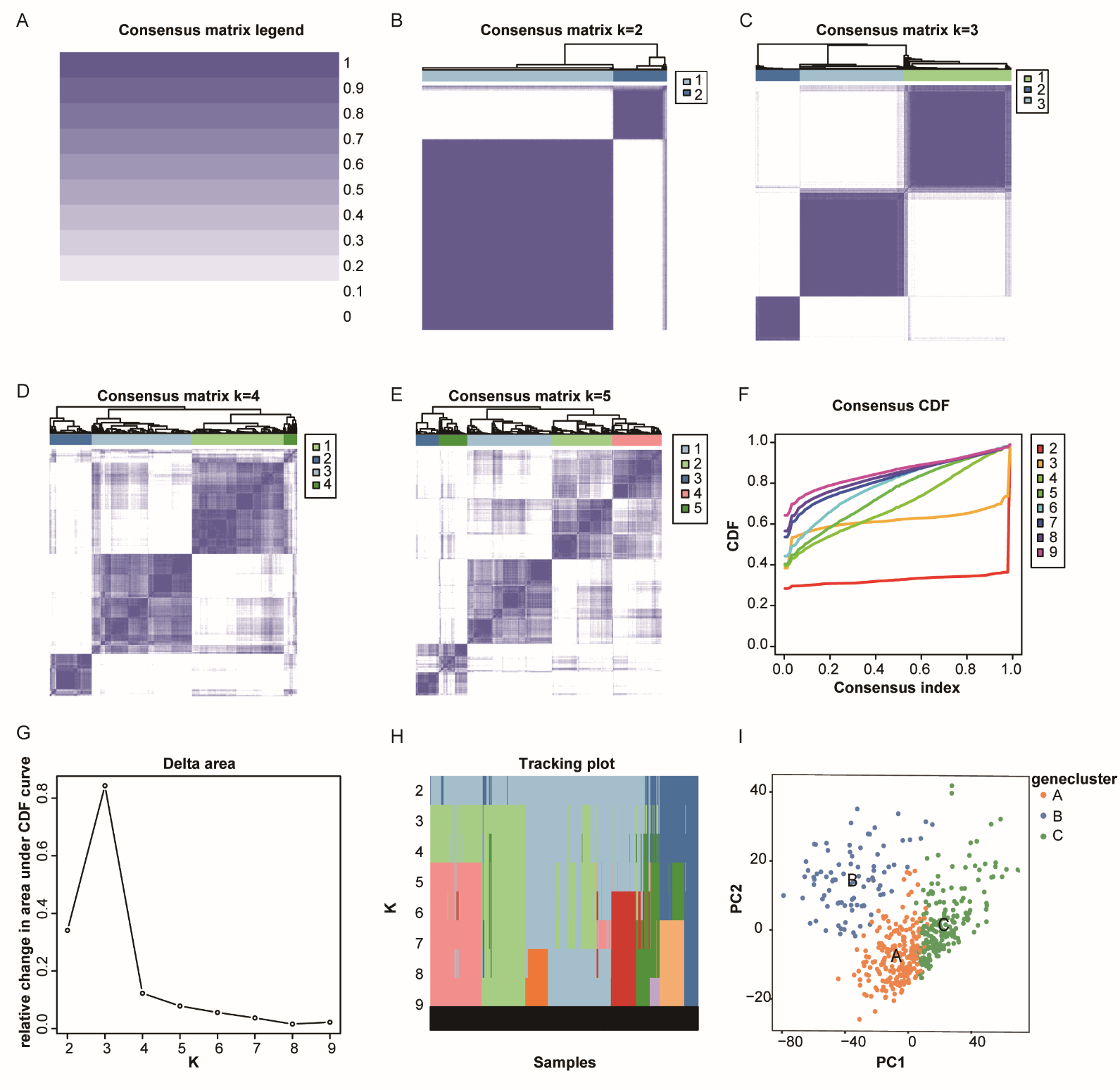


Supplementary Figure 4. Unsupervised clustering for OS-related DEGs. (A-E) TCGA-LGG cohort was grouped into 3 clusters according to the consensus clustering matrix (k = 3). (F) Uniform clustering cumulative distribution function (CDF) with k from 2 to 9. (G) The change of area under CDF curve with k from 2 to 9. (H) The tracking plot showed the relationship between samples and clusters. (I) The PCA plot showed the distribution among 3 gene clusters. OS, overall survival; DEGs, differentially expressed genes; TCGA, the Cancer Genome Atlas; LGG, lower-grade glioma (WHO Ⅱ and Ⅲ); CRGs, cuproptosis related genes; CDF, cumulative distribution function; PCA, principal component analysis.


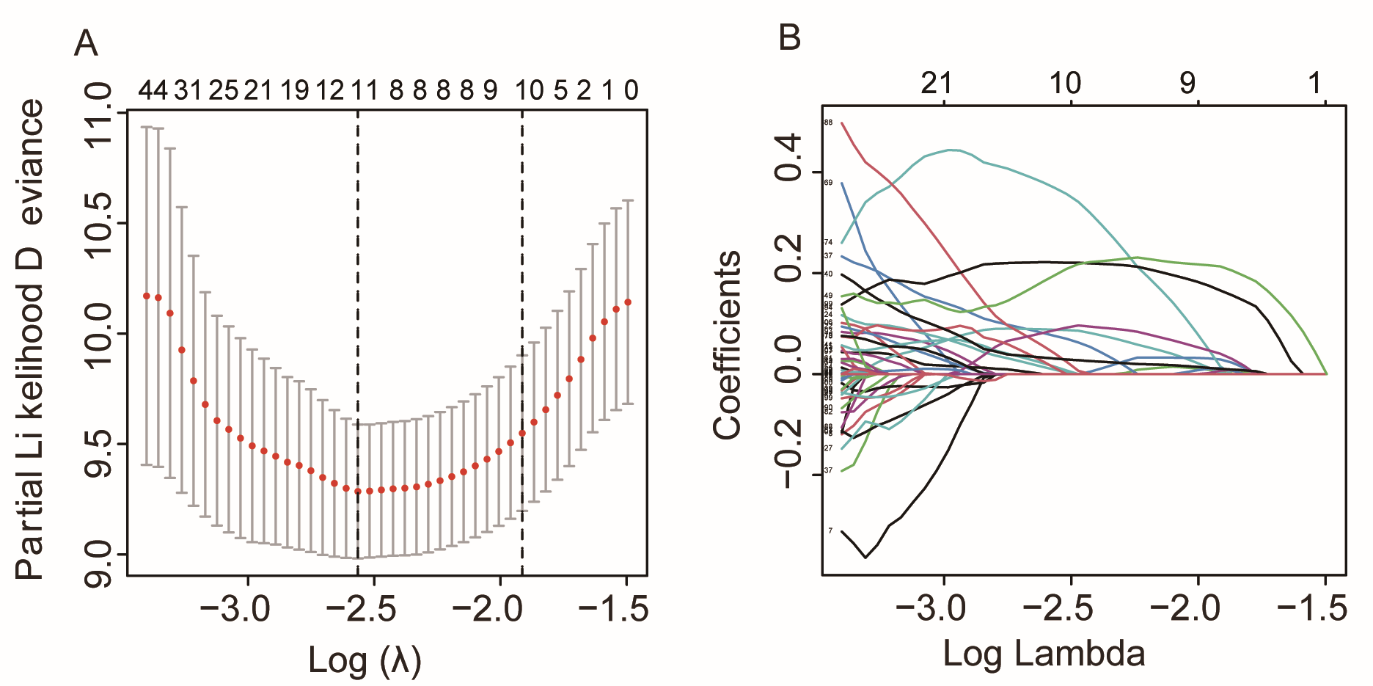


Supplementary Figure 5. LASSO coefficient profiles of OS-related DEGs and cross-validation for tuning the parameter selection in the LASSO regression. LASSO, least absolute shrinkage and selection operator. OS, overall survival; DEGs, differentially expressed genes.


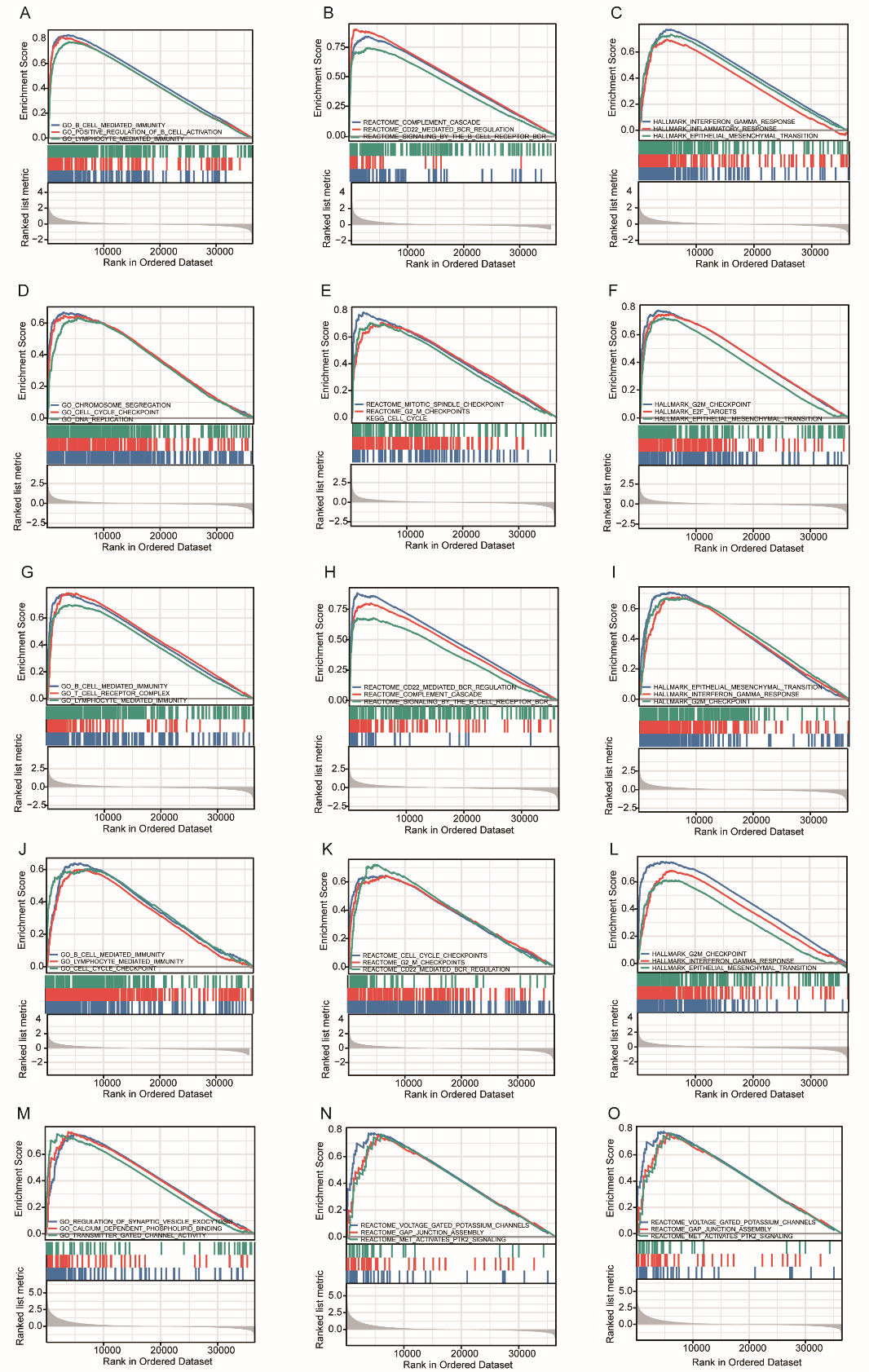


Supplementary Figure 6. Functional annotation of 5 genes in cuproptosis-related prognostic model. (A-C) GSEA between *C21orf62* high- and low-expression samples. (D-F) GSEA between *DRAXIN* high- and low-expression samples. (G-I) GSEA between *ITPRID2* high- and low-expression samples. (J-L) GSEA between *MAP3K1* high- and low-expression samples. (M-O) GSEA between *MOXD1* high- and low-expression samples. GSEA, Gene Set Enrichment Analysis.


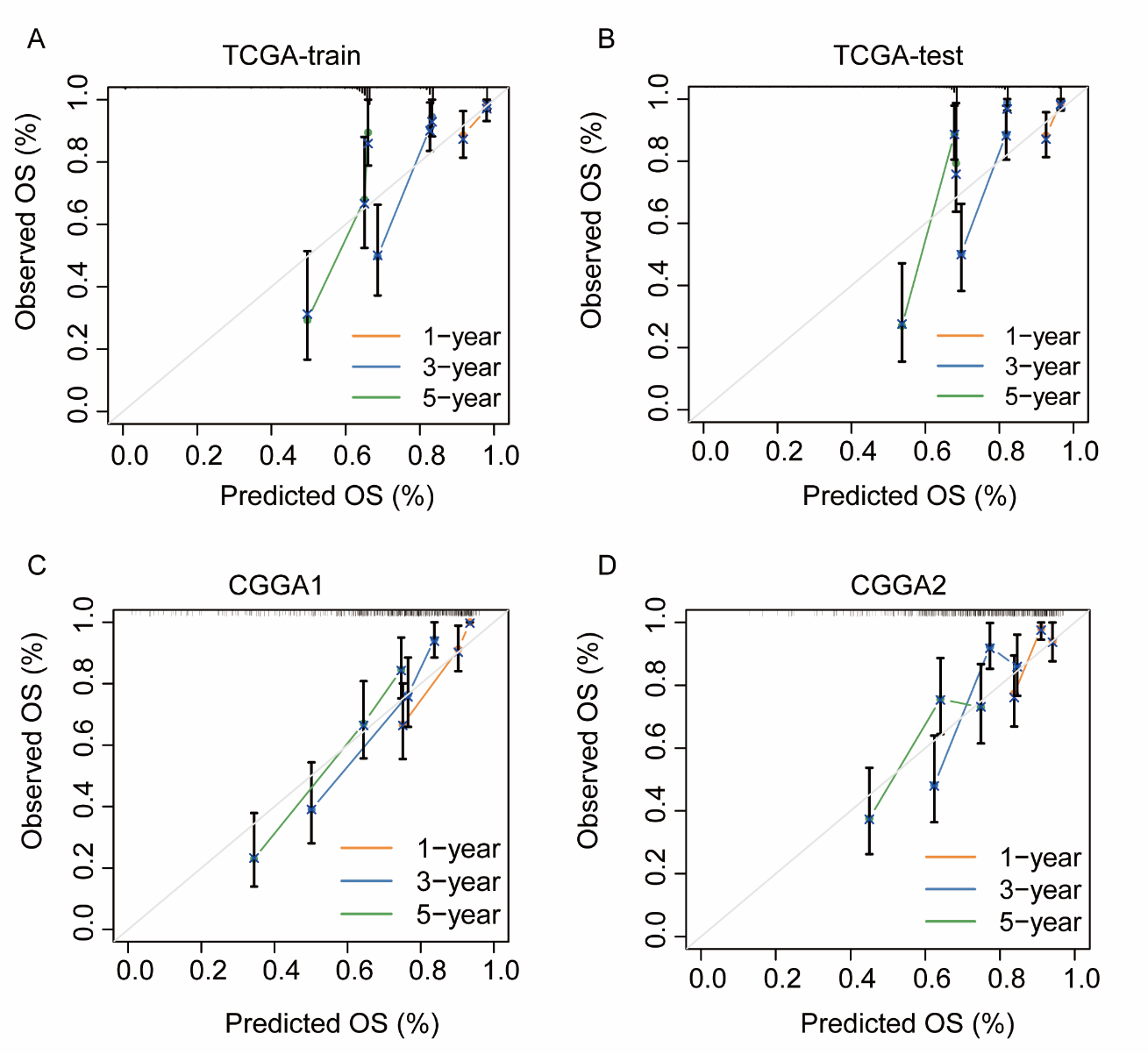


**Supplementary Figure7.** Calibration curves of the CRG_score for predicting probabilities of 1-, 3-, and 5-year survival. (A-B) Calibration curves of the CRG_score for predicting probabilities of 1-, 3-, and 5-year survival based on TCGA-training and TCGA validation cohorts. (C-D) Calibration curves of the CRG_score for predicting probabilities of 1-, 3-, and 5-year survival based on CGGA1 cohort and CGGA2 cohort.


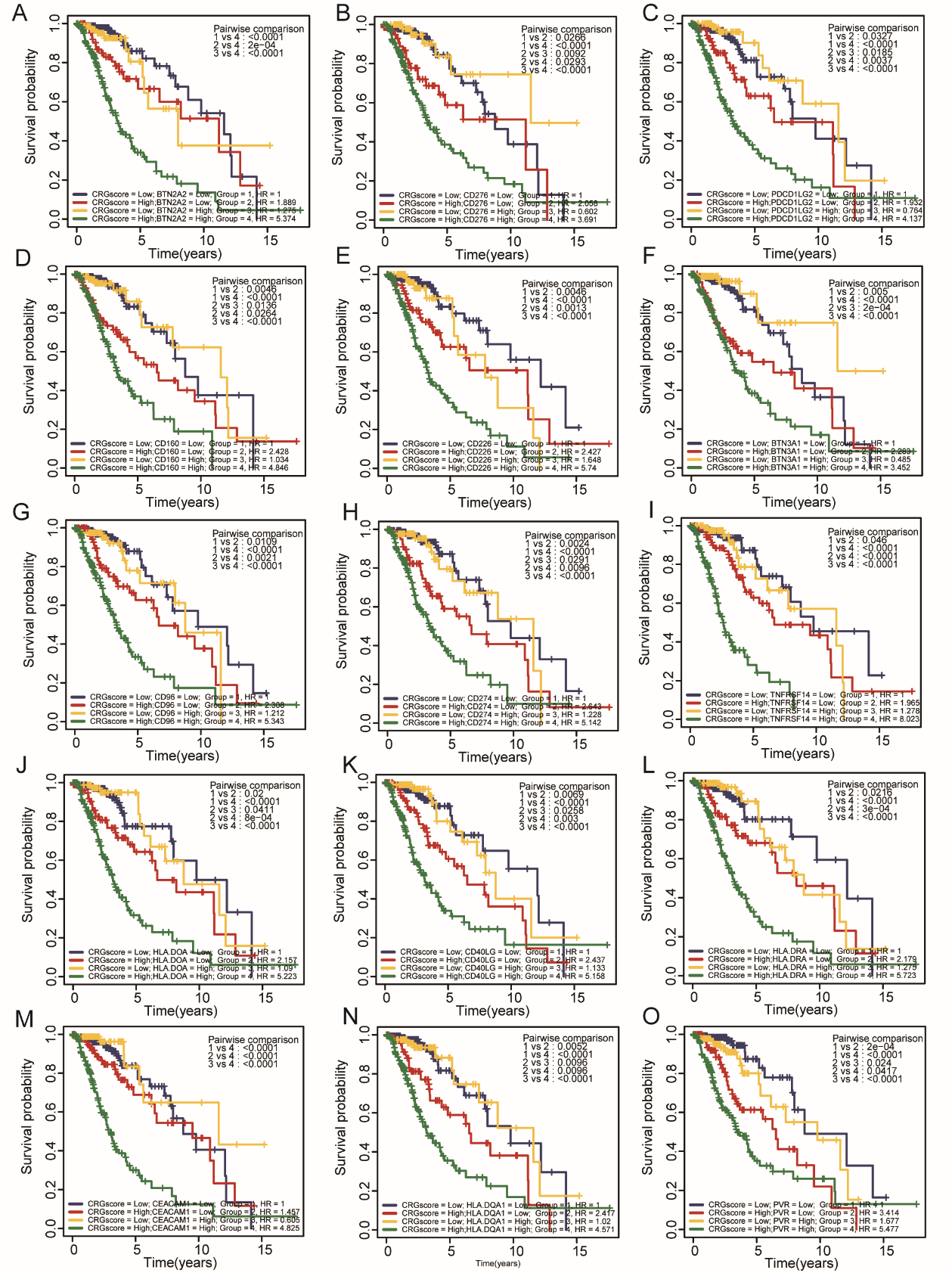


Supplementary Figure 8. The Kaplan–Meier OS curves among 4 groups classified by CRG score and the expression levels of (A) *BTN2A2,* (B) *CD276*, (C) *PDCD1LG2*, (D) *CD160*, (E) *CD226*, (F) *BTN3A1*, (G) *CD96*, (H) *CD274*, (I) *TNFRSF14*, (J) *HLA-DOA* (K) *CD40LG,* (L) *HLA-DRA* (M) *CEACAM1* (N) *HLA-DQA1* and (O) *PVR* in TCGA-LGG cohort. OS, overall survival; CRGs, cuproptosis related genes.


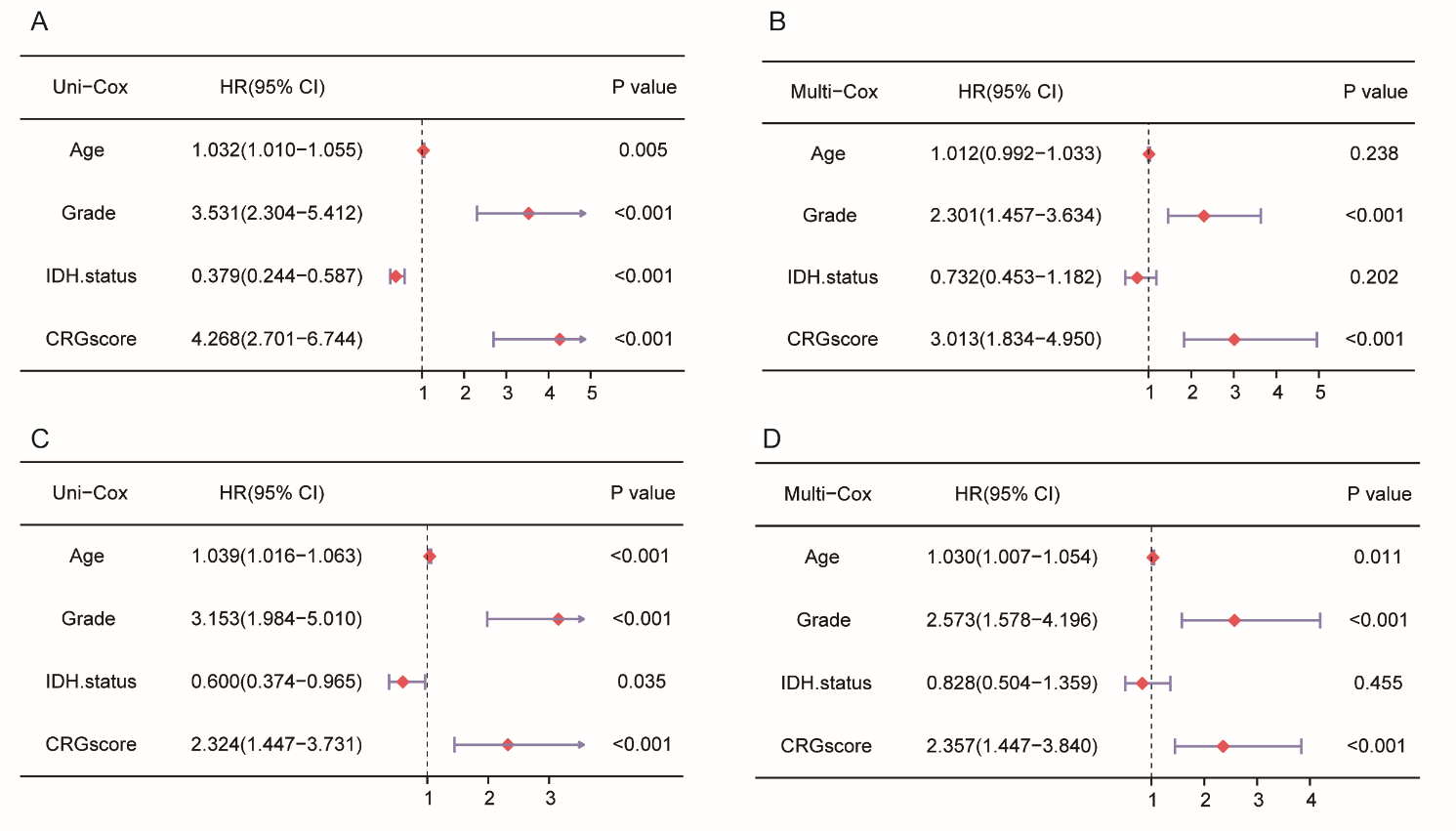


Supplementary Figure 9. Independent prognostic analysis in CGGA1 and CGGA2 cohorts. (A) Univariate Cox regression analysis of CRG score and clinical characteristics in CGGA1 cohort. (B) Multivariate Cox regression analysis of CRG score and clinical characteristics in CGGA1 cohort. (C) Univariate Cox regression analysis of CRG score and clinical characteristics in CGGA2 cohort. (D) Multivariate Cox regression analysis of CRG score and clinical characteristics in CGGA2 cohort. CGGA, Chinese Glioma Genome Atlas; CRGs, cuproptosis related genes.

## Supplementary Tables

Supplementary Table 1. Summary of 13 cuproptosis related genes.

Supplementary Table 2. Clinical characteristics of patients enrolled.

Supplementary Table 3. Oligo sequences used in quantitative real-time PCR.

Supplementary Table 4. The activation states of GOBP and KEGG terms between CRG clusters by GSVA.

Supplementary Table 5. GSEA of significant HALLMARK between CRG clusters.

Supplementary Table 6. Functional annotation of DEGs between two CRG clusters.

Supplementary Table 7. The characteristics of CGGA1 and CGGA2 cohort.

Supplementary Table 8. GSEA of significant GOBP and HALLMARK terms between high- and low-risk group.

Supplementary Table 9. Correlations between the expression of ICGs and CRG score.

Supplementary Table 10. Comparison between different models.
